# Enhancing Food Safety in the Cold Chain Through Internet of Things and Artificial Intelligence

**DOI:** 10.1111/1750-3841.70871

**Published:** 2026-02-16

**Authors:** Wagner Augusto Müller, Sandro Binsfeld Ferreira, Suse Botelho da Silva

**Affiliations:** ^1^ Coldtag Tecnologia da Informação LTDA São Leopoldo Brazil; ^2^ Unisinos University São Leopoldo Brazil; ^3^ University College Dublin Dublin Ireland

**Keywords:** artificial intelligence, food industry, food monitoring, food safety, Internet of Things

## Abstract

Effective cold chain management is crucial for ensuring food safety, but traditional monitoring approaches remain reactive and fragmented. Industry 4.0 technologies, such as the Internet of Things (IoT) and artificial intelligence (AI), offer transformative potential for real‐time tracking, automation, and predictive analytics. Many studies have explored the application of IoT and AI in the cold chain, but the existing literature focuses on isolated technologies. This review presents an integrated perspective, examining the synergistic potential of IoT‐enabled data acquisition and AI‐driven predictive analytics in the cold chain. We reviewed 97 peer‐reviewed papers published between 2010 and 2025, following PRISMA guidelines, covering IoT sensors, AI applications, and their integration across the food cold supply chain. Our analysis reveals a rapid growth in IoT and AI adoption, driven by regulatory and consumer demands for transparency, quality, and predictive risk assessment. IoT sensors enable real‐time monitoring, providing early detection of potential safety risks. AI‐powered models process sensor data to predict temperature deviations, assess food safety, and optimize logistics, reducing spoilage and contamination risks. We also highlight current limitations and future research directions, such as the limited number of studies on closed‐loop systems, where IoT sensors provide real‐time data and AI models respond dynamically. This review provides a comprehensive resource for selecting IoT and AI systems to enhance food safety and ensure a more resilient, transparent, and sustainable cold chain.

## 1 Introduction

The Food Waste Index Report 2024 by the United Nations Environment Programme (2024) highlights that in 2022 alone, 1.05 billion tonnes of food were wasted, representing 19% of all food available to consumers across retail, food service, and households. Perishable products that rely on cold chain (CC) logistics are disproportionately affected. For example, fruits and vegetables alone account for about one‐third of avoidable food waste in Europe, with meat, fish, and dairy also contributing substantially (Uhlig et al. 2025). In developing regions, limited infrastructure exacerbates this problem; in sub‐Saharan Africa, 30%–50% of produce is lost due to insufficient CC capacity and management (Mmereki et al. 2023). In this sense, failures during handling and transfers are critical points where temperature deviations accelerate deterioration, resulting in substantial economic losses (Damdam et al. 2023; Kumar and Sharma 2024), wasted environmental resources (Alfian et al. 2017), and increased risk of microbial contamination, which contributes to approximately 600 million foodborne illnesses and 420,000 deaths globally each year (World Health Organization 2024).

The challenge of maintaining CC integrity is further complicated in modern global supply chains spanning across continents, where temperature, humidity, and handling practices vary widely. (Haider et al. 2024; Baralla et al. 2021). To further compound this problem, monitoring remains mostly fragmented, mainly due to the lack of real‐time tracking and reliance on manual checks or periodic sampling, resulting in a reactive rather than proactive approach that delays responses to failures (Kumar and Sharma 2024). Additionally, when temperature abuses occur, there is limited predictive capability to assess their impact on product shelf life. This often leads to Type I failures (discarding a safe product unnecessarily) or Type II failures (allowing a contaminated product to reach consumers).

Over the past 15 years, the Fourth Industrial Revolution (Industry 4.0) has reshaped the way companies operate, accelerating the shift toward data‐driven decision‐making to boost efficiency, safety, and compliance. Technologies such as the Internet of Things (IoT) and artificial intelligence (AI) enable automation, predictive analytics, and end‐to‐end traceability. These tools can also enhance various aspects of the CC, including reducing food loss (Afreen and Bajwa 2021), improving safety and traceability (Baralla et al. 2021), and optimizing transportation routes and energy costs (Nakandala et al. 2016), shifting the food industry toward reactive, resilient, and sustainable supply chains.

This paper provides a systematic review of the potential of Industry 4.0 technologies, particularly IoT and AI, to enhance CC safety. Although prior reviews (e.g., Zou et al. (2014); Badia‐Melis et al. (2018); Loisel et al. (2021); Zhong et al. (2023); Feng et al. (2023); Mustafa et al. (2024)) have provided valuable perspectives, most are either dated, do not include recent advances, address only one technological dimension (AI or IoT), or adopt a broad scope that overlooks the specific nuances of CC logistics. Given the rapid evolution of these technologies, new advancements and applications are emerging at an unprecedented pace. This review aims to bridge the gap between former publications by offering an updated and focused perspective on how IoT and AI are revolutionizing CC management, enhancing traceability, automation, and predictive analytics for improved food safety and quality.

## 2 Methods

### 2.1 Search and Selection Process

A systematic literature review was conducted across three databases: Scopus, ScienceDirect, and Web of Knowledge. Only peer‐reviewed journal articles published between 2010 and February 2025 were included, while conference proceedings and symposium papers were excluded. For IoT‐related studies, the search combined the keywords “IoT” OR “Internet of Things” with “cold chain” OR “food temperature”, whereas for AI‐related studies, “artificial intelligence” OR “machine learning” was combined with “cold chain.”

### 2.2 Inclusion and Exclusion Criteria

The inclusion criteria included studies focusing on AI and IoT with explicit relevance to food CC, published in peer‐reviewed journals, and written in English. The exclusion criteria were: studies on Industry 4.0 applications in other sectors (e.g., healthcare); studies in the food sector with different scopes (e.g., crop or soil optimization); non‐English publications; conference proceedings and symposium papers; and duplicate entries.

### 2.3 Data Analysis

**FIGURE 1:**
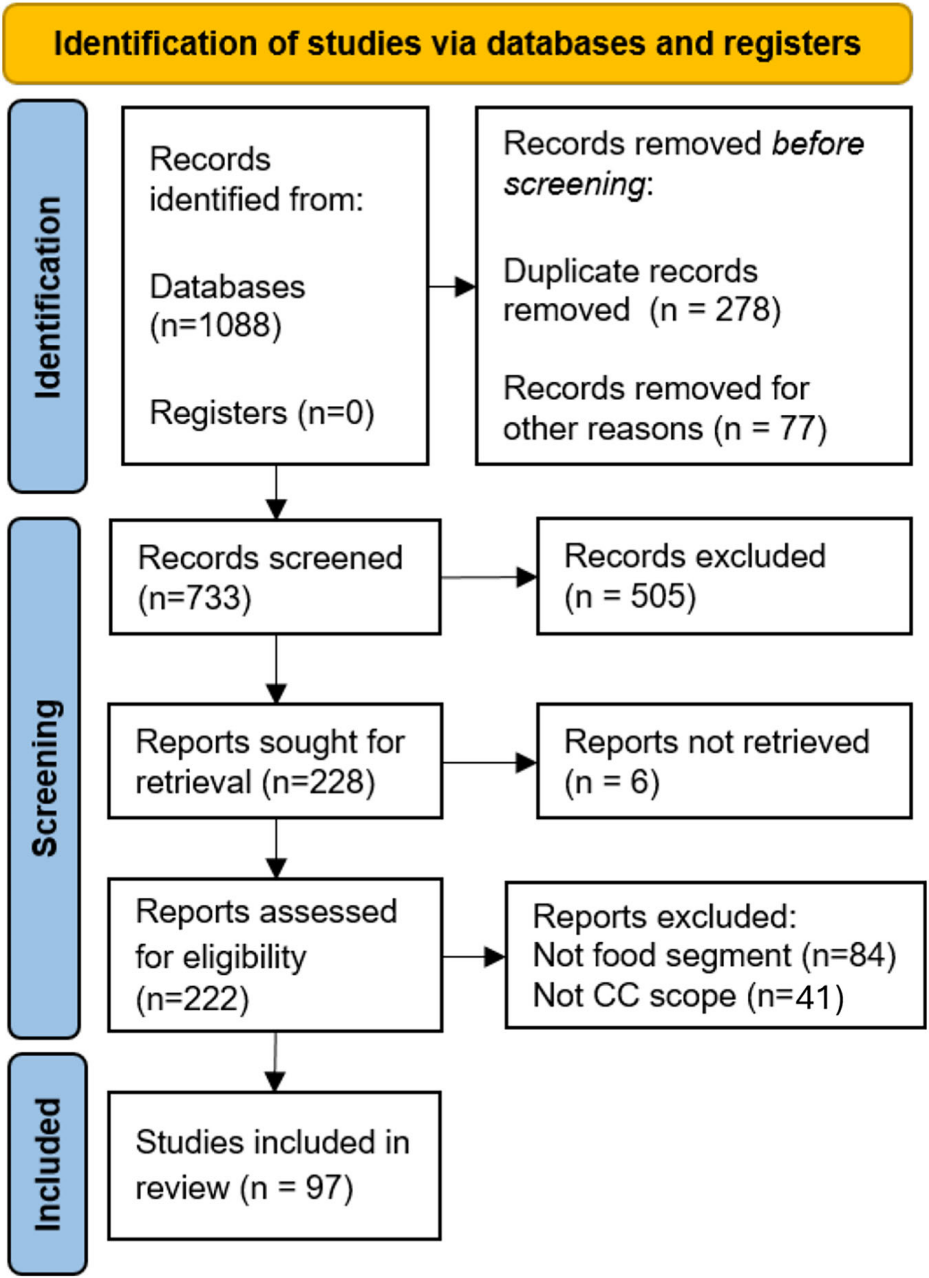
PRISMA flow diagram of the analyzed data.

The data analysis involved reading all selected papers and cataloging key information from each study. Studies were grouped by topic: IoT‐focused, AI‐focused, with studies addressing both included in both categories. Extracted data included the study title, year of publication, objectives, types of IoT sensors applied (if any), specific foods monitored (if applicable), AI algorithms used (if any), key findings, and reported challenges. The data were synthesized by categorizing sensors according to food applications, comparing the objectives of various AI models, analyzing current challenges and limitations, and identifying directions for future research. The review findings were reported following PRISMA (preferred reporting items for systematic reviews and meta‐analyses) guidelines (Figure 1), with Table 1 summarizing the number of papers included and excluded at each stage for both IoT and AI. Key results were organized into thematic sections covering IoT trends, AI applications, and current challenges.

**TABLE 1:** Quantitative summary of studies excluded in each step for IoT and AI.

Processing step	IoT	AI	Total
Initial records	766	322	1088
Duplicates removed	−184	−94	−278
Abstracts not written in English	−52	−25	−77
Records removed after abstract screening (non‐English, symposium/conference papers, not relevant)	−379	−126	−505
Full‐text not available	−2	−4	−6
Excluded after full‐text review (not in food segment or outside CC scope)	−91	−34	−125
**Final studies included**	58	39	97

## 3 Results

### 3.1 IoT

IoT integrates the Internet with smart objects such as sensors, network components, electronics, and RFID tags, enabling real‐time tracking. In CC logistics, it enables real‐time monitoring of environmental parameters, helping to prevent spoilage, reduce waste, and optimize operations. An IoT‐based system operates in multiple layers, encompassing real‐time data collection, transmission, processing, and visualization. Detailed coverage of IoT architectures is outside this paper's scope; thorough analyzes of IoT layers are provided elsewhere (e.g., Dauda et al. (2024); Mrabet et al. (2020)).

#### 3.1.1 Trends

**FIGURE 2:**
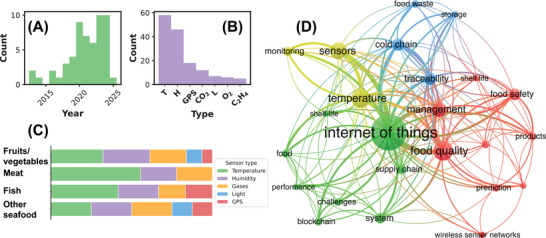
(A) Temporal evolution of IoT‐related publications within the CC context. (B) Most frequently analyzed sensors: temperature (T), humidity (H), and light (L). (C) Frequency of sensor usage in studies targeting specific products. (D) Co‐occurrence network of IoT‐ and CC‐related terms.

Figure 2A presents the evolution of IoT‐related publications in CC over time. The earliest publications identified were from 2013, and the upward trend highlights IoT's expanding role in food supply chains, particularly after 2018, aligning with the broader adoption of Industry 4.0 in the food sector over the last years (Alsubai et al. 2024; Feng et al. 2024). The slight decrease in publications in 2021 and 2022 may be partly related to disruptions caused by the COVID‐19 pandemic, although this explanation remains hypothetical. Since the literature search was completed at the end of February 2025, results from this year are only partially captured.

Figure 2D presents the keyword co‐occurrence network from IoT studies. Each node represents a keyword, with its size indicating the frequency of publications in which it appears. Keywords that frequently co‐occur are grouped and color‐coded, revealing thematic clusters. The most prominent word beyond **IoT** is **temperature**, a critical parameter for **monitoring** **food quality**, which shows a direct link to the **IoT** central keyword. Other central drivers of **IoT** adoption in the **CC** context, such as **food waste**, **traceability**, and **food safety**, also emerge clearly, underscoring their importance in ongoing research. Emerging technologies, as **blockchain** and **prediction** algorithms, appear as additional nodes that enhance **monitoring** and automation within the **CC** ecosystem. Therefore, the growing interest in IoT reflects a research agenda influenced by well‐established drivers in other segments of the food industry, including food safety (Alfian et al. 2020), sustainability (Baghel et al. 2024), and consumer demand for freshness and traceability (Gillespie et al. 2023).

Figure 2B summarizes the most frequently used sensors, while the complete list of studies and their applied sensors is provided in Supporting Information. Temperature sensors were present in all 58 studies, highlighting their central importance in CC monitoring and corroborating their prominent position in the co‐occurrence network. Beyond temperature, the most common sensors were **humidity**, **GPS**, and **CO_2_**. Humidity sensing is vital for preventing moisture‐related damage and microbial growth, especially in products sensitive to condensation or dryness. Many temperature sensors (e.g., DHT11) also include humidity measurement, which explains their frequent joint use and contribution to maintaining product quality.

CO_2_ sensors are important for monitoring air quality within CC environments, where elevated concentrations may indicate poor ventilation or increased microbial activity. For fresh produce, CO_2_ levels reflect respiration dynamics: sudden rises indicate accelerated oxygen consumption, leading to faster deterioration. In vacuum‐packaged products, controlled CO_2_ levels suppress microbial growth and delay ripening, linking gas composition to product quality and safety, key themes identified in the co‐occurrence network. **GPS** sensors, in turn, provide real‐time location data to ensure route compliance, enhance security, and support route optimization (see Section 3.2.2), thereby reducing transit times for perishable goods.

Research on CC monitoring includes both general frameworks applicable to all products and studies tailored to the specific needs of individual perishables. Figure 2C presents the distribution of sensor types across the four most frequently studied food categories identified in the literature. Since temperature sensors were present in all studies, their bar length was normalized to 100%. The size of the remaining bars should therefore be interpreted relative to temperature. For instance, a category reaching half the length of the temperature bar indicates that half of the studies also employed that sensor. Coloured bars represent the proportional use of humidity, gas, light, and GPS sensors, revealing distinct monitoring strategies across product categories.

Fruits and vegetables were the most studied category, with ten papers addressing issues such as microbial spoilage, premature ripening, wilting, and rooting. Their continuous CO_2_ release and ethylene formation (a gas accelerating ripening and the seventh most monitored sensor (Figure 2B)) justify the frequent use of gas sensors. **Light** sensors were also common, reflecting the need to control illumination to avoid discolouration, chlorophyll loss, or sprouting. By contrast, meat and fish were each examined in five studies, typically using temperature, humidity, and gas sensors. In meat, inadequate temperature control promotes the growth of pathogens such as *Salmonella* spp. and *E. coli* O157:H7, increasing the risk of foodborne illness (Peng et al. 2024; Pessoa et al. 2021). Fish deterioration, on the other hand, is accelerated by both microbial activity and histamine formation, which may cause food poisoning (Zhang et al. 2021; Pessoa et al. 2021). For both categories, maintaining strict temperature control remains the primary strategy to ensure food safety and quality.

Reliable IoT data transmission in the CC depends on appropriate IoT communication protocols. Reported technologies include USB (Kumar and Sharma 2024), Bluetooth (Alfian et al. 2017), LoRa (Qian et al. 2022), cellular networks (3G–5G) (Roduit et al. 2019), and satellite communication (Maheswaran et al. 2024), each suited to specific contexts. In warehouses, **Wi‐Fi** typically suffices due to existing infrastructure, whereas mobile scenarios require broader coverage. **Cellular networks** offer high throughput for moving vehicles, while **satellite links** ensure connectivity in remote or oceanic regions, albeit at a higher cost. **LoRa** enables low‐power, long‐range communication for minimal‐data tasks such as pallet‐level tracking. Protocol selection ultimately involves trade‐offs between coverage, bandwidth, energy use, and cost, with hybrid systems often providing the most efficient balance.

This subsection outlined trends in the reviewed literature on CC monitoring, covering publication dynamics, monitored variables, food categories, and communication protocols. Temperature‐centric sensing dominates, while secondary sensors (humidity, CO_2_, ethylene) are chosen according to the specific product being monitored. This focus reflects the central role of temperature as the most safety‐critical parameter; however, it also suggests that studies without product‐specific gas or volatile monitoring may overlook initial indicators of biochemical spoilage. IoT devices form the technological foundation of current monitoring practices; yet integration with complementary tools is increasingly essential for converting raw sensor data into actionable intelligence, which is the focus of the next subsection.

#### 3.1.2 Integration With Other Tools

IoT applications still face challenges such as data loss, manipulation, and security breaches, raising concerns over privacy and regulatory compliance. Sensor malfunctions, calibration errors, or connectivity failures may also yield inaccurate data, undermining decisions and product quality (Ahmad et al. 2024). This section examines recent IoT integrations with complementary technologies aimed at mitigating these issues, while unresolved gaps are further discussed in Section 4.1.

A major challenge in IoT systems is the risk of data manipulation, loss or inaccessibility. Blockchain offers a promising solution by replacing centralized data management with a decentralized ledger, making unauthorized modifications virtually impossible. Each transaction is time‐stamped and immutable, ensuring traceability in the food sector (a key aspect highlighted in Section 3.1.1). Consequently, **blockchain** appeared in several reviewed IoT studies and is itself visible in the co‐occurrence network (Figure 2D) linked to the **challenges** node, underscoring its relevance for data security and transparency in CC systems. Specific applications include using blockchain to reinforce consumer confidence by securing temperature and quality data (Grecuccio et al. 2020; Singh and Raza 2023; Yu et al. 2025). For instance, Qian et al. (2022) integrated blockchain into a temperature‐monitoring system estimating viral viability, strengthening trust under pandemic conditions. Similarly, blockchain‐based frameworks were proposed to optimize food‐quality data management through index value analysis (Alsubai et al. 2024) and to enhance reliability and data consistency in smart tourism services (Baralla et al. 2021). Further implementations have targeted product‐specific CC chains, including shellfish (Feng et al. 2020), fish (Grecuccio et al. 2020; Feng et al. 2024), and frozen aquatic products (Zhang et al. 2021).

Traditional IoT systems rely heavily on sensor batteries. Beyond the risk of power depletion, battery disposal also raises significant environmental concerns. Energy harvesting offers a sustainable alternative by converting ambient energy, such as vibrations or thermal gradients, into usable power for sensors, eliminating the need for conventional batteries. For instance, a self‐powered food monitoring system that uses far‐field radio frequency energy harvesting combined with temperature sensors and deep learning techniques to assess product quality was developed (Do et al. 2021). Similarly, a self‐powered RFID temperature monitoring system, which employs piezoelectric energy harvesting to convert mechanical vibrations from transportation into electrical energy, was also suggested (Chu et al. 2013). This demonstrates the feasibility of using transportation movements as an energy source for IoT‐based food monitoring. Likewise, the MS1089A nano‐power temperature sensor, which incorporates energy harvesting capabilities and enables ultra‐low power consumption, significantly extends battery life and reduces the need for frequent replacements (Maheswaran et al. 2024).

A key challenge for decision‐makers is how to transform IoT data into actionable insights. Integrating IoT with statistical process control (SPC) offers a practical solution. SPC uses statistical tools, particularly control charts, to monitor process variability and maintain quality within predefined limits. When combined with real‐time IoT data, it enables precise tracking of critical parameters such as temperature and energy use, supporting cost and sustainability goals in CC operations. For example, Montes et al. (2024) applied Shewhart, EWMA, and CUSUM charts to detect deviations in temperature and energy consumption, introducing a performance indicator to link both variables and identify inefficiencies in real time. Likewise, Shih and Wang (2016) integrated wireless sensor networks with control charts to monitor temperature during braised pork distribution, optimizing energy use by alternating between frozen and cool storage. Both studies showed that IoT‐based SPC systems can reduce spoilage, lower energy costs, and improve profitability, underscoring their potential for smart CC management. However, in terms of interpreting IoT data, one of the most promising avenues is its integration with AI. While SPC offers a structured approach to monitoring IoT data, integrating IoT with AI opens far broader possibilities. AI can analyze complex sensor data in real time for applications that go well beyond the scope of traditional SPC, and the next section will explore these applications in greater depth.

Integrating IoT with blockchain, SPC, and edge analytics helps address key challenges of data integrity, automated control, and real‐time anomaly detection. The studies indicate that blockchain enhances traceability and consumer trust when paired with IoT inputs; yet it also increases system complexity and cost. This creates a paradox where the most secure solutions remain least accessible to smaller operators. Meanwhile, energy‐harvesting shows strong potential for low‐maintenance monitoring, though commercial readiness and standardization remain limited. To bridge this gap between laboratory promise and operational deployment, future efforts should emphasize interoperability standards, cost–benefit analyzes and incremental pilot projects.

### 3.2 AI Trends and Integration With IoT

**FIGURE 3:**
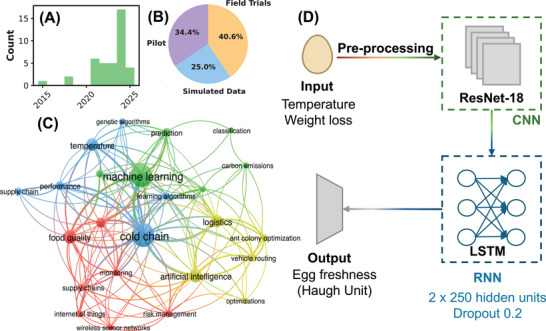
(A) Temporal evolution of IA‐related publications within the CC context. (B) Maturity stages of the analyzed studies, including pilot, field trials, and simulated data. (C) Co‐occurrence network of IA‐ and CC‐related terms. (D) Schematic of a hybrid neural network architecture, adapted from Kim et al. (2022).

AI is a branch of computer science that develops algorithms capable of performing tasks traditionally associated with human intelligence, such as learning from data, identifying patterns, and making decisions. While a detailed review of AI algorithms is beyond the scope of this paper, interested readers may consult classical sources such as Russel and Norvig (2009). In CC management, AI has been applied to optimize efficiency, minimize waste, and preserve product quality during storage and transportation. Figure 3A shows the evolution of AI‐related publications in CC, indicating a notable increase after 2021, likely associated with advances in predictive analytics, IoT integration, and optimization models. The peak around 2024 reflects strong momentum in AI‐driven research, possibly linked to post‐pandemic emphasis on supply chain resilience and sustainability in temperature‐sensitive logistics (Ramirez‐Asis et al. 2022).

Figure 3C illustrates a co‐occurrence network of keywords highlighting the role of AI in CC logistics. **Machine learning** emerges as the most central keyword, linking all major clusters, including research on **algorithms** and **prediction**. It is also closely connected to the second most prominent term, **cold chain**, underscoring the pivotal role of AI in this domain. Other key terms, such as **food quality**, **risk assessment**, and the **Internet of Things**, further highlight how AI‐driven technologies are applied to maintain optimal storage conditions, anticipate potential risks, and prevent foodborne hazards. Notably, the yellow cluster, which focuses on the intersection of AI and **logistics optimization**, reflects a growing trend toward using AI for **vehicle routing**.

Figure 3B illustrates the readiness level distribution across all analyzed studies. Field trials account for the largest share (50%), indicating a shift from development toward application. The rest are split between pilot‐scale implementations (42%) and simulated data approaches (31%), with some overlap due to hybrid studies (e.g., Kim et al. (2022)). Pilot studies often test more complex sensing strategies, such as bioimpedance or volatile compound monitoring (Huang et al. 2023b), which remain difficult to deploy outside controlled settings. By contrast, field‐deployed technologies have primarily focused on temperature, reflecting the relative ease of embedding such sensors into logistics systems. Therefore, simulated and pilot studies push methodological frontiers, while field trials consolidate feasibility. It may be emphasized that the substitution of complex, messy real‐world data with simulated/sanitized datasets can fail to replicate critical conditions, such as the thermal heterogeneity within a multi‐compartment refrigerated vehicle, the impact of fluctuating traffic, or unpredictable door opening frequency. The reliance on synthetic data can create a “generalization gap,” where models achieve inflated performance metrics in controlled settings but suffer dramatic accuracy losses upon real‐world deployment. This indicates the need for extensive field testing to ensure that the reported efficacy is maintained under operational duress.

In general, various AI algorithms can be applied, ranging from simple regression models to advanced deep learning architectures. Figure 3D illustrates a complex example: a hybrid neural network model from Kim et al. (2022), combining convolutional neural networks (CNNs) for feature extraction and pattern recognition (e.g., images or structured signals) with long short‐term memory (LSTM) networks for sequential or time‐series modeling. The model was trained on 2750 samples (1850 from static/dynamic storage and 900 from real CC conditions). To address data scarcity, augmentation expanded the dataset to 6875 training samples, with 1375 reserved for testing. Training parameters (optimizer = Adam, learning rate = 0.001, batch size = 64, epochs = 25–50) were tuned via Bayesian optimization using RMSE as the objective function.

At the data acquisition layer, the system collects storage temperature and egg weight loss (analogous to the perception layer in an IoT architecture) with temperature loggers providing continuous time‐series data and digital scales tracking gradual mass changes. Real CC data were sampled daily. Because raw data are noisy and irregular, preprocessing includes filtering (moving average), normalization, and interpolation to align sequence lengths. In the intelligence layer, the hybrid model operates: the CNN (ResNet‐18) extracts discriminative features from sensor signals, which feed into a stacked bidirectional LSTM (two layers, 250 hidden units each, dropout = 0.2) to capture temporal dependencies and reduce overfitting.

Finally, at the decision layer, the model predicts the Haugh Unit—a standard egg freshness index—with strong performance (RMSE = 2.02, MAE = 1.40, *R* ^2^ = 0.96), outperforming simpler regressors. This example demonstrates a complete IoT–AI pipeline: from sensor‐level acquisition and preprocessing to data augmentation, training, and inference for actionable freshness prediction. While this case highlights the structure and features of a hybrid architecture with optimization, dropout and augmentation, non‐hybrid models (CNNs, RNNs, ANNs) and even simpler regressors have also shown excellent performance. Ultimately, architecture choice depends on application complexity, data availability and computational constraints.

Table 2 summarizes the analyzed studies, presenting the applied models, objectives, and reported accuracy (A), root mean square error (RMSE) and *R* ^2^ values (when available). Most models achieved accuracies above 90%, low RMSE and *R* ^2^ values close to 1, indicating a strong goodness‐of‐fit. When compared with classical models, such as the Arrhenius equation for shelf‐life estimation, AI consistently showed superior predictive. Nonetheless, these metrics should be interpreted cautiously, considering factors such as interpretability, computational cost, and data requirements, which may favor simpler models in some contexts. For instance, studies relying on linear or polynomial regression models (e.g., Fatorachian and Pawar (2025); Jackson et al. (2024)) provide fully transparent relationships between temperature, time, and demand, which facilitate regulatory auditing and root‐cause analysis in food safety investigations. In contrast, neural networks achieve superior accuracy but often show limited interpretability and therefore require additional mechanisms to support accountability.

**TABLE 2:** Ranges of conditions and product characteristics for the datasets used in each model. Statistical indices can be reported as a function of variables, for example, RMSE(T) for temperature, RMSE(t) for time, and RMSE(G) for gas.

Study	Model(s)	Goal	Metrics
Haider et al. (2024)	Whale optimization and extreme learning machines (ELM)	Temperature estimation and measure product quality	*A* = 99.83%
Nakandala et al. (2016)	GA, fuzzy GA, and SA	Logistics optimization	
Feng et al. (2024)	LSTM, particle swarm optimization and SVM	Product quality	*A* = 93.3%; RMSE = 0.014; R^2^ = 0.98
Peng et al. (2024)	Logistic regression, SVM, RF, AdaBoost, and XGBoost	Safety estimation	A = 80.0%
Pessoa et al. (2021)	GBM	Measure product quality	A(meat type) = [50 − 70]%
Kanjilal et al. (2025)	Multiple linear regression, polynomial regression, random forest (RF), support vector regression, Gaussian process regression, LSTM	Measure product quality	RMSE(T,G) = [0.03 − 0.20]; R^2^(T,G) = [0.96 − 0.99]
Fatorachian and Pawar (2025)	ARIMA and multiple linear regression	Demand forecast	RMSE = 0.06
Jackson et al. (2024)	Seasonal ARIMA	Demand forecast	
Loisel et al. (2021)	Multilayer perceptron regressor (MLP)	Temperature estimation	RMSE(position) = [0.5 − 1.5]℃
Zou et al. (2023)	Back‐propagation (BP), long short‐term memory (LSTM), and deep NN	Temperature estimation	RMSE(position) = [0.24 − 0.62]℃
Ktenioudaki et al. (2021)	Boosted regression tree	Measure product quality	R^2^ = 0.84; RMSE = 0.84
M and Voola (2025)	RF, decision trees (DT), K‐nearest neighbors (KNN), support vector machine (SVM), Catboost	Measure product quality	A = 98.9%
Zakeri et al. (2018)	DT, artificial NN (ANN)	Measure product quality	A = 91.2%
Wang et al. (2024)	Genetic algorithm (GA)	Measure product quality	A ≥ 93%
Nong et al. (2024)	Linear regression, DT, SVM, feedforward NN, RF, and gradient boosting machine (GBM)	Equipment performance	R^2^=[0.98 ‐ 0.99]
Kim et al. (2022)	Convolutional neural network (CNN) and LSTM	Measure product quality	RMSE = 2.02
Chen et al. (2024)	BP, support vector regression, KNN, and RF	Measure product quality	R^2^=0.98; RMSE = 0.01
Liu et al. (2024a)	Modified ant colony optimization (MACO)	Logistics optimization	
Liu et al. (2024b)	GA, MACO and simulated annealing (SA)	Logistics optimization	
Xu et al. (2024)	Deep reinforcement learning	Equipment performance	
Eze et al. (2024)	KNN	Temperature estimation	
Shi (2024)	KNN	Logistics optimization	RMSE = 2.9889
Tang et al. (2021)	DT, SVM, weighted KNN, and ensemble methods	Equipment performance	A ≥ 95%
Yang et al. (2024)	DACO and adaptive golden section search	Logistics optimization	
Hu et al. (2021)	MACO	Logistics optimization	
Abdella et al. (2021)	General AI models	Temperature estimation	
Zhang et al. (2024b)	CNN, BP	Measure product quality	A = 97%
Meng et al. (2024)	BP	Alarm optimization	A = 98.6%
Zhang et al. (2024a)	SVM, linear discriminant analysis, KNN, and RF	Measure product quality	R^2^(fruit) = [0.95 − 0.99]
Huang et al. (2023a)	BP, RBF, SVM, and ELM	Measure product quality	Accuracy = 94.0% R^2^(T) ≥ 0.99 RMSE(T) = [0.036 − 0.077]
Huang et al. (2023b)	ELM, SVM, BP, and RBF	Measure product quality	A = 91.6% R^2^(T) = [0.97 ‐ 0.99] RMSE(T) = [0.07 − 0.10]
Xia et al. (2023)	SVM and ANN	Measure product quality	
Yuan et al. (2025)	MLP	Sustainability	A = 99.6% RMSE = 0.07
Guo et al. (2022)	2‐dimensional MACO	Logistics optimization	
Hu et al. (2024)	Least absolute shrinkage and selection operator	Quality estimation	A(T) = [72‐91]%
Lim et al. (2022)	Mayfly and ELM	Product temperature	A(T) = [0.91–0.92] R^2^(T) = [0.91 ‐ 0.92] RMSE = [0.17 − 0.22]
Lau et al. (2021)	ANN	Risk assessment	
Park et al. (2023)	MLP	Temperature estimation	R^2^(t,T,U) = [0.90 − 0.92]
Arabsheybani et al. (2024)	Support vector clustering and data‐driven robust optimization	Logistics optimization	

Most models developed in the analyzed studies employed relatively shallow architectures, like a small number of neurons and layers, which make them computationally efficient and readily applicable in modern devices. From a food safety perspective, computational cost is not merely a technical concern but a determinant of system reliability. Models that cannot run continuously on edge devices or that require frequent cloud connectivity introduce latency and single points of failure, which are incompatible with real‐time hazard prevention.

Across studies, three macro‐patterns are visible. (1) Methodological diversity: AI applications span simple regressions to complex architectures, reflecting both diverse problem types (forecasting, estimation, classification) and uneven data availability. (2) A performance–validation gap: many models report excellent lab metrics, but limited availability of large, representative field datasets and few large‐scale deployments, producing a generalization gap when models move from controlled to operational settings. (3) Emphasis on applied optimization and decision‐support: recent work increasingly targets routing, energy use and alarm reduction rather than pure detection, showing an orientation toward operational value.

Scalability emerges as a decisive differentiator among the reviewed AI approaches. Models requiring high‐frequency data streams, extensive preprocessing, or GPU‐based inference (e.g., deep CNN–LSTM architectures) may be well suited for centralized analytics but remain challenging to deploy across large, heterogeneous cold‐chain networks. In contrast, lighter‐weight models such as regression, decision trees, and selected ensemble methods demonstrate greater scalability, as they can be executed on edge devices or embedded controllers, enabling broader adoption across small‐ and medium‐sized operators. It is important to emphasize that the viability of AI models extends beyond predictive performance, requiring evaluation frameworks that also consider robustness, interpretability, and scalability. Overall, the analyzed AI models can be grouped into four categories: regression, classification, optimization, and neural networks (NNs).

Regression‐based models are best suited for CC applications where transparency, stability, and low computational overhead are dominant requirements. They perform reliably under limited or structured data conditions and are particularly appropriate for demand forecasting, capacity planning, and compliance‐oriented monitoring, where regulatory acceptance and human interpretability outweigh marginal gains in predictive accuracy. Classifiers occupy an intermediate position between classical regressors and deep learning models. They offer higher predictive accuracy than linear models while retaining partial interpretability through feature importance analysis. This makes them well suited for quality classification, safety risk estimation, and equipment performance monitoring in operational CC environments characterized by noisy or incomplete IoT data.

NNs achieve the highest predictive accuracy in complex, nonlinear tasks such as freshness estimation and temperature prediction. However, their reliance on large datasets, higher computational demands, and limited interpretability restrict their direct use in highly regulated or resource‐constrained environments. Consequently, they are best deployed as decision‐support or upstream inference components feeding optimization or rule‐based control systems. Optimization algorithms address a different problem class: decision optimality rather than prediction accuracy. Their suitability lies in routing, scheduling, and multi‐objective trade‐offs involving cost, perishability, emissions, and service level constraints. These models do not require large labeled datasets but depend instead on well‐defined objective functions and constraint formulations. In CC contexts, optimization models are most effective when paired with predictive models that estimate dynamic inputs such as spoilage rates or demand uncertainty.

Table 3 shows the comparative suitability of different AI models, and highlights that no single model class dominates across all dimensions: regression and ensemble models prioritize transparency and robustness, NNs maximize predictive accuracy, and optimization algorithms deliver superior decision optimality under complex operational constraints. Effective cold chain systems therefore require hybrid architectures that combine prediction, optimization, and human oversight. Specific application for each class will be discussed in the next subsections.

**TABLE 3:** Comparative suitability of AI model classes.

Model	Predictive accuracy	Decision optimality	Data requirements	Interpretability	Regulatory fit
Regression	Low–medium	Low	Low	High	High
Classification	Medium–high	Low	Moderate	Medium	Medium–high
NNs	High	Low	High	Low	Low–medium
Optimization	N/A	High	Moderate	Medium	Medium

#### 3.2.1 Regression Models

Regression models are effective for predicting continuous outcomes and identifying trends in data. While other methods usually achieve superior performance, regression models can present promising results in certain contexts while being less computationally demanding. Notably, two studies on demand forecasting in CC logistics applied regression techniques and reported interesting results. ARIMA and MLR have been applied to capture seasonal demand and assess the influence of factors such as temperature and promotions (Fatorachian and Pawar 2025). The study highlighted that IoT‐enabled real‐time monitoring of temperature and humidity improved predictive accuracy and reduced waste, increasing both safety and sustainability in CC logistics. In contrast, a second study introduced a customer segmentation approach using segmented ARIMA, which improved forecasting accuracy and optimized warehouse capacity, helping identify underutilized freezer space at a warehouse (Jackson et al. 2024). Interestingly, in the former study, the ARIMA‐MLR model outperformed an ML alternative, proving to be particularly effective for capacity planning and large‐scale efficiency optimization.

#### 3.2.2 Optimization Algorithms

Optimization algorithms are widely applied to routing problems, as seen in the co‐occurrence network (Figure 3C). Routing optimization approaches differ based on the factors considered. Guo et al. (2022) targeted multi‐compartment vehicle routing to reduce fuel costs and CO_2_ emissions, while Liu et al. (2024b) extended this to electric vehicles by incorporating charging, cooling, and time‐window constraints. Real‐time traffic congestion was addressed by Yang et al. (2024), who optimized single‐compartment routing with traffic and carbon emissions in mind. Other studies have considered fuel and refrigeration costs, cargo damage, and soft time‐window penalties (Hu et al. 2021), while recent work explores flying deliveries accounting for epidemic risks, environmental impact, and UAV types (Liu et al. 2024a). NNs and classifiers can also support optimization algorithms by preprocessing data. For example, clustering sellers by proximity improves distribution efficiency (Shi 2024), or constructing uncertainty sets from historical data to enable robust routing under variable conditions (Arabsheybani et al. 2024).

Although all reviewed studies target the CC segment, they rarely account for product‐specific perishability. Minimizing route costs and travel time can reduce exposure to temperature fluctuations, but perishability rates vary across products and ideally should be incorporated into optimization models. Nakandala et al. (2016) is a notable exception, prioritizing faster delivery for highly perishable goods to align routing with product‐specific degradation. However, even this study considers perishability only at the category level, without accounting for the history of individual products. In reality, perishability depends not only on the product type but also on its environmental history, such as temperature profile, highlighting the need for models that integrate degradation functions, which still represent a significant research gap. NNs could, for instance, track individual product deterioration in closed‐loop systems (see Section 4.4). Additionally, stochastic delivery factors such as fluctuating demand, traffic variability (as done by Yang et al. (2024)), and unexpected disruptions point to the need for adaptive optimization. While most CC studies rely on static models, dynamic approaches emerging in other sectors (Ajayi 2023) could offer a promising avenue for more effective route optimization.

#### 3.2.3 Classifiers

Classification algorithms were widely applied in predictive modeling for tasks such as quality evaluation and logistics/equipment performance.

*Quality*: Classifiers have been applied to assess Product quality. For instance, banana ripeness was predicted based on temperature, humidity, and O_2_/CO_2_ levels, categorizing samples as “ripe,” “not ripe,” or “spoiled” (M and Voola 2025). Similarly, oysters were classified into fresh, sub‐fresh, and decayed categories using ambient and sensory evaluations (Wang et al. 2024). Other examples include predicting pig meat inspection outcomes based on animal welfare conditions (Pessoa et al. 2021), evaluating salmon freshness through bioimpedance signals (Zhang et al. 2024a), and estimating blueberry weight loss from humidity and temperature measurements (Ktenioudaki et al. 2021). Beyond direct quality assessment, classifiers can also support food safety monitoring. A recent study applied classification algorithms to analyze *Salmonella* prevalence in retail chicken supply chains, revealing significant variations across different supply modes (Peng et al. 2024). The model successfully identified key genetic markers associated with contamination, illustrating the potential of classifiers for epidemiological surveillance and contamination risk prediction. When integrated with advanced genomic analyzes, these approaches offer powerful tools for enhancing safety management in cold supply chains.

*Logistics/equipment performance*: Classification algorithms can also support logistics and CC operations. For example, they can be used to determine optimal temperature ranges for preserving different products (Eze et al. 2024). Beyond product‐specific guidance, classifiers can enhance equipment performance within the CC. In this context, they have been applied to predict door status (open or closed) based on temperature signals, helping to reduce unnecessary door openings and improve both energy efficiency and food safety (Tang et al. 2021). Similarly, classification techniques have been used to model non‐linear relationships in CC devices, such as the correlation between equipment temperature and heat transfer coefficients, enabling optimization of system performance (Nong et al. 2024).

#### 3.2.4 Neural Networks

NNs are inspired by the human brain and can be applied throughout CC logistics for prediction, decision support, scalability, and detecting critical events such as alarms (Ramirez‐Asis et al. 2022). Although temperature is a key factor in food spoilage, 98% of recorded temperatures in refrigerated vehicles exceeded the recommended range (setpoint ± 0.5 ℃), revealing the limits of traditional monitoring (Zou et al. 2023). Using IoT sensor data (e.g., ambient temperature, humidity, loading/unloading events, door status), AI models outperform rule‐based approaches in temperature estimation. For instance, NNs achieved 99.83% accuracy in monitoring tomato temperatures using only ambient and humidity data (Haider et al. 2024). Likewise, combining air temperature and GPS data with thermal simulations improved pallet temperature predictions, with models trained on experimental data outperforming those using synthetic data (Loisel et al. 2021). Consistent findings have been reported across multiple studies (Abdella et al. 2021; Lim et al. 2022; Zou et al. 2023; Park et al. 2023)

Beyond temperature estimation, most NN applications focus on quantifying **food quality**, as also reflected in its prominence within the AI co‐occurrence network (Figure 3C). In this context, NNs can be trained with ambient conditions and product characteristics at time t=0 to predict its properties at a later time dt. By continuously monitoring food freshness, these models help optimize storage conditions, reduce food waste, and enhance logistics efficiency, representing a significant advancement in smart food safety aspects. These approaches typically leverage the ability of NNs to excel at processing complex data, making them particularly well‐suited for such predictive tasks.

NN applications in product quality are typically product‐specific. For instance, egg quality has been predicted from weight loss and temperature (Kim et al. 2022), milk quality from tank temperature and level (Zakeri et al. 2018), lamb meat freshness from temperature–impedance signals (Huang et al. 2023b), and tilapia freshness from temperature, humidity, volatile nitrogen, and microbial counts (Feng et al. 2024). In all cases, NNs achieved strong predictive performance (see Table 2). Similar to IoT‐based monitoring trends (Section 3.1.1), most NN studies also target fruits and vegetables. For example, environmental parameters (humidity, temperature, and O_2_/CO_2_) have been used to predict weight loss and firmness in cucumbers and strawberries (Zhang et al. 2024b), with similar methods applied to grapes (Chen et al. 2024), bananas (Kanjilal et al. 2025), and blueberries (Huang et al. 2023a).

NNs can also optimize alarm and control systems in CC logistics. For example, instead of triggering notifications based solely on temperature, a NN can generate alarms using multiple inputs, including compartment and ambient temperatures, initial food temperature, door status, pre‐cooled state, and cumulative door‐open time (Meng et al. 2024). Initial food temperature and cumulative door‐open time were the most influential factors for accurate recognition. Additionally, NNs can optimize cooling water circulation in refrigeration systems, achieving more stable and precise regulation than traditional control methods (Xu et al. 2024).

Some studies have explored less conventional NN applications within the CC domain. For instance, NNs have been used for risk quantification, incorporating inputs such as temperature, monitoring errors, regulatory deviations, and workforce turnover to predict risks related to equipment failures, operational inefficiencies, market uncertainties, and personnel issues (Lau et al. 2021). This approach demonstrated strong adaptability and practical relevance in an Australian CC case study. Moreover, NNs combined with econometric models were used to evaluate policy effectiveness and promote the sustainability of distribution networks (Yuan et al. 2025). By integrating factors such as policy type, number of policies issued, and effectiveness scores, the models predicted a Logistics Development Score and forecasted future CC scenarios under varying policy interventions, offering valuable insights for sustainable logistics planning.

It can be seen that some contradictions occur across algorithm classes. First, optimization and routing algorithms frequently assume accurate perishability models or perfect IoT inputs; in practice, sensor drift, intermittent connectivity, and heterogeneous device standards introduce noise and missingness that significantly affect optimizer behavior. Second, many AI architectures are tuned for a single commodity (eggs, tilapia, bananas), limiting transferability across food classes. Practically, these contradictions imply some priorities for the field: (a) produce and share multi‐vendor, multi‐region datasets and evaluation benchmarks to reveal real‐world robustness; (b) pair algorithmic advances with XAI and formal audit trails to satisfy regulators and practitioners; and (c) design modular, commodity‐aware models that allow composable reuse (e.g., base models for temperature‐driven spoilage plus product‐specific modules for intrinsic attributes). Meeting these priorities will be essential to move AI from excellent lab demonstrations to trustworthy, scalable CC systems.

This section reviewed AI applications in the food CC, covering general insights such as the growing interest in the field, potential applications, and specific approaches reported in the literature. With the current state of the art in both IoT and AI outlined, the next section examines their integration in greater depth, while highlighting ongoing challenges and research gaps in the field.

## 4 Discussion and Current Challenges

This section examines trends emerging from the 97 reviewed papers, highlighting key patterns, synergies, and current limitations in applying AI and IoT within the food CC. As shown in Figures 2 and 3, research interest in both technologies has steadily increased in recent years. Although the food industry is often considered slower to adopt digital innovations than high‐tech sectors such as automotive or aerospace (Sharma et al. 2023; Romanello and Veglio 2022), the gap is even more pronounced when compared with the pharmaceutical CC. In the pharmaceutical sector, advanced digital monitoring and analytics systems are already mitigating temperature‐related product failures; some studies report that over 75% of temperature‐excursion incidents can be avoided using Industry 4.0 tools in the segment (ASHP 2021; Minhar 2025). Market analyzes further show that global investment in CC monitoring technologies is substantial, projected to rise from USD 15.89 billion in 2023 to USD 55.75 billion by 2030 in the pharmaceutical industry (GVR 2024). IoT‐enabled traceability tools are also increasingly used for real‐time tracking of vaccines and biologics (Wang 2023; Kumari 2025).

While food products generally suffer quality degradation or financial loss when the CC is breached, pharmaceutical goods (such as vaccines) operate under a zero‐tolerance regime: even a 1–2 ℃deviation can render a shipment unusable or unsafe (Minhar 2025; ASHP 2021). This heightened risk threshold has driven “bullet‐proof” compliance in pharma, supported by stringent regulations and real‐time monitoring infrastructures. This cross‐industry context reveals that, despite the different risk profiles, the food sector can still draw valuable lessons from pharmaceutical cold chains, particularly regarding traceability, interoperability, and resilience to last‐mile disruptions.

It is important to note that, although IoT and AI both fall under the Industry 4.0 framework, they play complementary and interconnected roles. IoT provides real‐time monitoring and data acquisition, while AI delivers analytical depth by transforming this data into actionable insights. This includes estimating product quality under varying environmental conditions, forecasting demand patterns, evaluating equipment performance, and assessing food safety risks. By identifying trends and anomalies, IoT‐enabled AI models facilitate a shift from reactive to predictive decision‐making, enabling earlier interventions, reduced spoilage and more efficient resource allocation.

The IoT‐AI synergy enables real‐time evaluation of product quality and safety, overcoming the limitations of static models that cannot capture the dynamic conditions of storage and transport. AI models trained on IoT time‐series data have effectively predicted tomato and banana ripeness by incorporating temperature, humidity, and handling factors (Haider et al. 2024; Kanjilal et al. 2025), reducing waste and safety risks. Likewise, IoT‐based GPS tracking combined with AI routing algorithms allows delivery routes to adapt dynamically to traffic and environmental conditions‐critical for perishable goods, where delays impact quality. The model by Nakandala et al. (2016), for instance, integrates perishability profiles into route planning, prioritizing deliveries by spoilage risk. Overall, IoT functions as the nervous system of the CC, collecting environmental data, while AI acts as the brain, interpreting signals and guiding decisions, creating a smarter, more resilient food industry. Despite these advances, challenges remain concerning IoT infrastructure, AI model development, and regulatory compliance, which are addressed in this section. Closed‐loop systems, the ultimate goal of IoT–AI integration, are also examined.

### 4.1 IoT Challenges

The growing deployment of IoT devices in the food CC has significantly expanded the system's attack surface, introducing new cybersecurity and data privacy challenges. Beyond traditional risks such as data theft, cyber‐physical attacks can actively manipulate sensor readings and cause physical harm. For example, a hacker could spoof a temperature sensor, transmitting false “safe” readings while perishable food deteriorates. The decentralized and heterogeneous nature of IoT networks further exacerbates vulnerabilities. Delayed firmware updates (where devices do not receive the latest software patches, leaving known vulnerabilities unaddressed) along with inconsistent device management, can leave devices unpatched and susceptible to exploitation (Kulkarni et al. 2024).

Addressing these risks requires a multi‐layered strategy that combines technological and procedural safeguards. Edge computing (processing IoT data closer to the source) reduces exposure to attacks while enabling real‐time threat detection and automated responses (Fotopoulos et al. 2022). Complementary measures such as data encryption, network segmentation, and multi‐factor authentication (Obaidat et al. 2020) enhance resilience by protecting sensitive information and limiting attackers' ability to move laterally across the network. Additionally, the reviewed studies indicate that blockchain technology is increasingly being explored to secure CC data, providing tamper‐proof records and transparent traceability, as discussed in Section 3.1.1. Together, these approaches can strengthen the integrity and confidentiality of IoT‐collected data against both conventional and sophisticated threats.

Looking forward, emerging cryptographic technologies promise to strengthen privacy and trust in IoT‐enabled CCs. Zero‐knowledge proofs allow stakeholders to verify data authenticity without revealing underlying details. For instance, a retailer can confirm shipment compliance without accessing its full history (Zhang et al. 2023). Homomorphic encryption enables AI models to analyze encrypted IoT data, such as consumer behavior or product conditions, without exposing raw information (Onoufriou et al. 2021). Beyond supply chain stakeholders, privacy implications extend to user‐level food tracking, where granular consumption or purchase histories could be linked to individuals. While such data streams enable personalized risk alerts and waste reduction strategies, they also raise concerns about surveillance, profiling, and unintended commercial exploitation. Future frameworks must balance transparency and personalization with strong privacy safeguards.

Beyond security, technical and operational challenges also constrain the reliability of IoT‐enabled CC. Sensor drift (gradual loss of calibration accuracy) can silently distort safety or shelf‐life assessments without regular recalibration or self‐correction trust (Oluwafemi et al. 2024). Interoperability remains another major barrier, as devices from different manufacturers often use incompatible protocols and data formats. This fragmentation hampers the integration of heterogeneous sensors into unified monitoring systems, limiting scalability and compliance. Addressing this issue requires harmonized data schemes and standardized middleware capable of bridging diverse IoT platforms.

### 4.2 AI Challenges

One of the primary challenges in deploying AI effectively in the CC is the scarcity of high‐quality, comprehensive datasets. Although many studies report AI applications, they often rely on limited, proprietary, or simulated data (see Figure 3B), which may not capture or be applied in the complexity of real‐world CC environments (Kang et al. 2019; Goyal and Mahmoud 2024). This can lead to numerical artifacts, biases, inaccuracies or AI hallucinations. Moreover, several models are developed using built‐in programming tools that function as “black boxes,” making it difficult to trace how specific decisions are made (Hassija et al. 2023). This lack of transparency poses significant challenges for regulatory compliance and accountability.

Across the reviewed literature, only a small subset of studies explicitly address how model decisions could be explained to regulators or auditors. Most deep learning—based quality or safety estimators, as NNs models, report performance metrics without providing traceable decision pathways, which limits their immediate suitability for regulated food safety environments. By contrast, tree‐based ensembles and regression models, although sometimes less accurate, align more naturally with existing food safety auditing practices due to their clearer decision logic. In high‐stakes applications such as predicting pathogen prevalence, accuracy alone is not enough, regulatory authorities also demand clear and transparent explanations for model outputs (Balta et al. 2025). The inherent opacity of complex models, particularly deep neural networks, creates a “trust deficit” in critical contexts. If an automated system commits a Type II error (e.g., allowing a contaminated product to pass inspection), the reasoning behind that decision must be fully auditable for legal and compliance purposes. One approach to addressing this challenge is the integration of XAI methods (Buyuktepe et al. 2023). These tools quantify the contribution of each input feature to the final prediction, transforming black‐box results into interpretable, traceable evidence. For example, they can show whether a spoilage prediction was driven by a gradual accumulation of minor temperature fluctuations or by a single, recent door‐opening event. Such transparent linkages are essential for establishing audit trails, ensuring regulatory compliance, and mitigating legal risk within the food sector.

Additionally, quality is influenced by both intrinsic and extrinsic parameters. However, the majority of current research focuses predominantly on environmental conditions (e.g., temperature, humidity, and gas concentration) neglecting intrinsic factors. For instance, egg quality is affected by the age of the laying hen (Şekeroǧlu et al. 2024); the animal's pre‐slaughter stress impacts meat quality through abnormal post‐mortem pH decline (Xing et al. 2018); and milk protein content varies with the breed and age of the cow (Cerbulis and Farrell 1975). As a result, approaches based on extrinsic factors are limited to predicting only part of the overall quality landscape. Efforts to capture intrinsic parameters, such as monitoring animal stress levels or physiological markers, are emerging in other research domains (Ogawa et al. 2024; Tatar 2024; Garlito et al. 2023; Papatsiros et al. 2024). Although a complete review of these measurements falls outside the scope of this review, incorporating such information into predictive models in the CC could significantly enhance the accuracy and reliability of product quality assessment systems. Integrating both intrinsic and extrinsic data represents a promising direction for the next generation of AI‐enhanced CC solutions.

The comprehensive integration of an Industry 4.0 framework also demands substantial initial capital investment (Dakhia et al. 2025). This financial barrier creates a pronounced technology adoption gap, often restricting implementation to large corporations. This can be mitigated if the technology demonstrates a clear and rapid return on investment (ROI). Early adopters of AI‐enabled supply chain management have reported reductions in logistics costs, improvements in inventory levels, and enhancements in service levels (Finmile 2025). Further financial analysis of AI platform implementation suggests potential returns on investment, with payback periods under 7 months (Cohen and Tang 2024). The deployment challenge, therefore, is not the absence of a financial case, but rather the difficulty in securing the initial capital required to overcome the high barrier to entry and demonstrate the system's viability at scale. Industry progress depends on developing modular, financially accessible entry points for smaller supply chain actors.

### 4.3 Regulatory Aspects

The reviewed literature reveals a fundamental trade‐off between predictive accuracy and trustworthiness, related attributes such as explainability, scalability, and computational feasibility. In food safety contexts (where automated decisions may trigger recalls, product disposal, or regulatory action) models must support not only accurate predictions but also transparent justification and reliable operation at scale. Yet, most studies prioritize accuracy in controlled settings, with fewer explicitly addressing how their systems would be audited, validated, or defended in real‐world regulatory scenarios.

Regulation in the food sector concerning AI and IoT is still in a developing stage, often struggling to keep pace with technological advancements. While food safety standards, such as those enforced by the FDA (U.S.), EFSA (EU), and Codex Alimentarius (international), focus heavily on temperature control, traceability, and contamination prevention, they do not yet address the deployment of AI‐driven decision systems or IoT infrastructure (Dakhia et al. 2025). IoT devices are increasingly covered under general data protection and cybersecurity laws (e.g., GDPR in Europe), but there is limited sector‐specific guidance for their application in CCs (Voigt and von dem Bussche 2024).

For example, the standard ISO 23412:2020 “Indirect, temperature‐controlled refrigerated delivery services‐Land transport of parcels with intermediate transfer” specifies operational requirements for refrigerated parcel delivery by land transport (ISO 2020). It focuses on the service provider's resources, operations and communications, but explicitly excludes direct refrigerated delivery services and does not cover the measurement of parcel‐contents temperature. Thus, while the certification may enhance operational credibility and traceability, it does not ensure audit‐trail integrity at the level required for electronic records under frameworks like 21 CFR Part 11 (which govern electronic records in regulated pharmaceutical/biomedical contexts). Therefore, organizations seeking legally defensible IoT data in high‐liability food‐safety situations must implement additional GxP‐style data protocols beyond ISO 23412's scope.

As for AI, regulatory frameworks are beginning to emerge; the EU's AI Act (Walters et al. 2023), for instance, establishes a risk‐based classification where food‐related AI applications may be deemed high‐risk if they directly affect public health or safety, imposing requirements for transparency, traceability, and human oversight. At the same time, many AI applications in food processing and CC logistics can fall under limited or minimal risk when framed as preparatory or decision‐support tools with documented human supervision. Globally, however, there remains no standardized regulatory blueprint governing the ethical, operational, and data‐handling aspects of AI–IoT integration in food supply chains. Moving forward, coordinated efforts between policymakers, technologists, and industry stakeholders will be essential to develop robust, harmonized standards that ensure safety, accountability, and consumer trust.

Although regulatory approaches across the Asia‐Pacific (APAC) region remain fragmented, they show visible alignment with risk‐based tiers and system testing, mirroring EU‐style requirements (Ho et al. 2024). Significant models include Singapore's Model AI Governance Framework and the AI Verify testing toolkit, which provides developers with a mechanism to assess and benchmark their AI systems against internationally recognized governance principles. In addition, regulatory agencies are also using AI to enhanced quality control. The FDA, for example, is leveraging AI to strengthen import screening and ensure food safety, focusing initially on imported seafood (Food and Administration 2022). The ML analyzes historical data to identify patterns unseen by human screeners, predicting the likelihood that an import shipment is harmful or non‐compliant with regulations.

### 4.4 Closed‐Loop Systems

Despite rapid advances in AI and IoT technologies, their current deployment in food CC management represents only a fraction of their potential. CC digital systems can be conceptually classified into three different levels depending on their actuation capabilities: (i) open‐loop monitoring systems, where IoT devices collect data without influencing operations; (ii) advisory systems, where AI models provide recommendations that are manually enacted; and (iii) fully closed‐loop or adaptive systems, in which sensing, inference, and automated actuation form a continuous feedback cycle. Most existing implementations fall into the first two categories, indicating that the systematic integration of IoT and AI is one of the primary gaps in current research. While progress toward such integration has been more evident in other sectors (Besigomwe 2025), persistent challenges (such as data integration difficulties, high upfront investment, cybersecurity vulnerabilities, and shortages of skilled personnel) continue to hinder adoption in food CCs.

The potential value of closed‐loop operation can be illustrated through real‐time shelf‐life management. IoT sensors can continuously track environmental variables such as temperature, humidity, and light exposure, while AI models dynamically update shelf‐life predictions as conditions evolve. These predictions can then inform timely operational responses, including targeted promotions for products nearing expiration or adjustments to storage conditions to slow degradation (Afreen and Bajwa 2021). However, realizing such responsiveness at scale requires moving toward systems capable of continuously translating predictions into action. From a systems perspective, this requirement frames closed‐loop CC management as a feedback control problem (Sharma 2017). IoT sensors provide real‐time measurements of the system state, AI models function as state estimators or predictors (Schöning et al. 2022), and control policies determine corrective actions such as refrigeration setpoint adjustments, routing changes, or alarm suppression. Compared to classical rule‐based control, AI‐enhanced feedback enables fast adaptation to the non‐linear, time‐varying, and partially observable conditions that characterize real CC environments. Yet, the effectiveness of such feedback critically depends on several aspects, such as sensor integration (e.g., support to legacy systems, connectivity, latency vs. bandwitdth), where and how decisions are executed, functional safety requirements, among other factors.

A recurring limitation in the reviewed literature is the reliance on centralized, cloud‐based inference, which introduces latency and connectivity dependencies that undermine real‐time control. Closed‐loop often requires edge intelligence, where inference and decision‐making occur close to the physical process (Singh and Gill 2023). Lightweight AI models deployed at the edge can support rapid corrective actions, while cloud‐based components retain a supervisory role for long‐horizon optimization, model retraining, and regulatory logging (Zhou and Chen 2025).

Digital twins provide an additional mechanism to bridge prediction and actuation. By maintaining a virtual representation of products, vehicles, or storage environments, digital twins enable the simulation and evaluation of control actions before their physical implementation (Kreuzer et al. 2024). Integrating IoT data streams with AI‐based degradation models allows operators to conduct ‘what‐if’ analyses (such as testing alternative routing strategies or refrigeration policies), thereby reducing operational risk in safety critical contexts (Radanliev et al. 2021).

Taken together, these considerations delineate a coherent set of architectural principles for closed‐loop IoT‐AI cold‐chain systems. Progress toward such systems will require coordinated advances in distinct areas, such as integrating product‐specific degradation models with real‐time control and routing decisions; developing hybrid human‐AI oversight mechanisms to ensure safety and accountability during the implementation of automated actuation and implementing adaptive strategies robust to latency, sensor drift, missing data, and distributional shifts.

## 5 Conclusion

This review shows that the convergence of Internet of Things and AI technologies is transforming the management of food cold chains. IoT has evolved from simple temperature tracking toward distributed, multi‐sensor systems capable of capturing complex environmental data in real time, while AI models increasingly convert these data into predictive insights for safety and logistics optimization. However, the two technologies have advanced unevenly: IoT deployments are growing in scope but remain fragmented by incompatible standards, whereas AI research demonstrates high analytical performance yet relies heavily on simulated data and limited field validation.

Across the literature, a clear movement emerges toward integrated IoT‐AI frameworks that can predict failures and adapt operations dynamically rather than reacting to deviations after they occur. Nonetheless, contradictions persist (laboratory accuracy not necessarily translates into real‐world robustness, the most powerful AI models often lack interpretability, and many “smart” systems still depend on manual oversight). These inconsistencies highlight that progress in this field depends not only on technological innovation but also on data availability, transparency, and system interoperability.

Looking ahead, the findings point to the need for standardized datasets, explainable and auditable AI, and sustainable sensor networks supported by energy‐efficient or self‐powered devices. Strengthening collaboration between researchers, industry, and regulators will be essential to close the gap between experimental prototypes and large‐scale deployment. Ultimately, the goal is to advance toward closed‐loop, self‐adaptive CCs where IoT data and AI analytics operate seamlessly to ensure safer, more transparent, and more sustainable food systems.

## Author Contributions

**Wagner Augusto Müller**: Conceptualization; investigation; writing – original draft; methodology; visualization; data curation; formal analysis. **Sandro Binsfeld Ferreira**: Conceptualization; funding acquisition; methodology; writing – review and editing; visualization; project administration; data curation; supervision; resources. **Suse Botelho da Silva**: Conceptualization; funding acquisition; methodology; writing – review and editing; visualization; project administration; data curation; supervision; resources.

## Conflicts of Interest

The authors declare no conflicts of interest.

## Supporting information



Table S1. Summary of sensors used in each study.

## References

[jfds70871-bib-0009] ASHP . 2021. Pharmaceutical Cold Chain Management Resource Guide. American Society of Health‐System Pharmacists.

[jfds70871-bib-0001] Abdella, A. , J. K. Brecht , and I. Uysal . 2021. “Statistical and Temporal Analysis of a Novel Multivariate Time Series Data for Food Engineering.” Journal of Food Engineering 298: 110477.

[jfds70871-bib-0002] Afreen, H. , and I. S. Bajwa . 2021. “An IoT‐Based Real‐Time Intelligent Monitoring and Notification System of Cold Storage.” IEEE Access 9: 38236–38253.

[jfds70871-bib-0003] Ahmad, K. , M. S. Islam , M. A. Jahin , and M. F. Mridha . 2024. “Analysis of Internet of Things Implementation Barriers in the Cold Supply Chain: An Integrated ISM‐MICMAC and DEMATEL Approach.” PLoS ONE 19, no. 7: e0304118. 38995917 10.1371/journal.pone.0304118 PMC11244810

[jfds70871-bib-0004] A Ajayi, O. 2023. “Real‐Time Route Optimization in Logistics: A Deep Learning Approach.” World Journal of Advanced Research and Reviews 19, no. 3: 1719–1722.

[jfds70871-bib-0005] Alfian, G. , M. Syafrudin , U. Farooq , et al. 2020. “Improving Efficiency of RFID‐Based Traceability System for Perishable Food By Utilizing IoT Sensors and Machine Learning Model.” Food Control 110: 107016.

[jfds70871-bib-0006] Alfian, G. , M. Syafrudin , and J. Rhee . 2017. “Real‐Time Monitoring System Using Smartphone‐Based Sensors and NoSQL Database for Perishable Supply Chain.” Sustainability 9, no. 11: 2073.

[jfds70871-bib-0007] Alsubai, S. , A. Alqahtani , A. Alanazi , and M. Bhatia . 2024. “Decision‐Tree‐Assisted Intelligent Framework for Food Quality Analysis.” IEEE Internet of Things Journal 11, no. 19: 30800–30807.

[jfds70871-bib-0008] Arabsheybani, A. , A. A. Khamseh , and M. S. Pishvaee . 2024. “Sustainable Cold Supply Chain Design for Livestock and Perishable Products Using Data‐Driven Robust Optimization.” International Journal of Management Science and Engineering Management 19, no. 4: 305–320.

[jfds70871-bib-0010] Badia‐Melis, R. , U. Mc Carthy , L. Ruiz‐Garcia , J. Garcia‐Hierro , and J. Robla Villalba . 2018. “New Trends in Cold Chain Monitoring Applications ‐ A Review.” Food Control 86: 170–182.

[jfds70871-bib-0011] Baghel, L. K. , R. Raina , S. Kumar , and L. Catarinucci . 2024. “IoT‐Based Integrated Sensing and Logging Solution for Cold Chain Monitoring Applications.” IEEE Journal of Radio Frequency Identification 8: 837–846.

[jfds70871-bib-0012] Balta, I. , J. Lemon , C. A. Popescu , et al. 2025. “Food Safety – the Transition To Artificial Intelligence (AI) Modus Operandi.” Trends in Food Science & Technology 165: 105278.

[jfds70871-bib-0013] Baralla, G. , A. Pinna , R. Tonelli , M. Marchesi , and S. Ibba . 2021. “Ensuring Transparency and Traceability of Food Local Products: A Blockchain Application To A Smart Tourism Region.” Concurrency and Computation‐practice & Experience 33, no. 1: e5857.

[jfds70871-bib-0014] Besigomwe, K. 2025. “Closed‐Loop Manufacturing with AI‐Enabled Digital Twin Systems.” Cognizance Journal of Multidisciplinary Studies 5, no. 1: 18–38.

[jfds70871-bib-0015] Buyuktepe, O. , C. Catal , G. Kar , Y. Bouzembrak , H. Marvin , and A. Gavai . 2023. “Food Fraud Detection Using Explainable Artificial Intelligence.” Expert Systems 42, no. 1: e13387.

[jfds70871-bib-0016] Cerbulis, J. , and H. Farrell . 1975. “Composition of Milks of Dairy Cattle. I. Protein, Lactose, and Fat Contents and Distribution of Protein Fraction.” Journal of Dairy Science 58, no. 6: 817–827. 1141480 10.3168/jds.S0022-0302(75)84644-3

[jfds70871-bib-0017] Chen, Q. , J. Li , J. Feng , and J. Qian . 2024. “Dynamic Comprehensive Quality Assessment of Post‐Harvest Grape in Different Transportation Chains Using SAHP‐CatBoost Machine Learning.” Food Quality and Safety 8: fyae007.

[jfds70871-bib-0018] Chu, H. , G. Wu , J. Chen , F. Fei , J. D. Mai , and W. J. Li . 2013. “Design and Simulation of Self‐Powered Radio Frequency Identification (RFID) Tags for Mobile Temperature Monitoring.” Science China Technological Sciences 56, no. 1: 1–7.

[jfds70871-bib-0019] Cohen, M. C. , and C. S. Tang . 2024. “The Role of AI in Developing Resilient Supply Chains.” Accessed November 3, 2025. https://gjia.georgetown.edu/2024/02/05/the-role-of-ai-in-developing-resilient-supply-chains/.

[jfds70871-bib-0038] Cold Chain Temperature Monitoring Market Size Report 2030 . 2024. Grand View Research. Accessed November 3, 2025. https://www.grandviewresearch.com/industry-analysis/cold-chain-temperature-monitoring-market.

[jfds70871-bib-0020] Dakhia, Z. , M. Russo , and M. Merenda . 2025. “AI‐Enabled IoT for Food Computing: Challenges, Opportunities, and Future Directions.” Sensors 25, no. 7: 2147. 40218659 10.3390/s25072147 PMC11991368

[jfds70871-bib-0021] Damdam, A. N. , L. O. Ozay , C. K. Ozcan , A. Alzahrani , R. Helabi , and K. N. Salama . 2023. “IoT‐Enabled Electronic Nose System for Beef Quality Monitoring and Spoilage Detection.” Foods 12, no. 11: 2227. 37297471 10.3390/foods12112227 PMC10252673

[jfds70871-bib-0022] Dauda, A. , O. Flauzac , and F. Nolot . 2024. “A Survey on IoT Application Architectures.” Sensors 24, no. 16: 5320. 39205014 10.3390/s24165320 PMC11360728

[jfds70871-bib-0023] Do, H.‐D. , D.‐E. Kim , M. B. Lam , and W.‐Y. Chung . 2021. “Self‐Powered Food Assessment System Using LSTM Network and 915 MHz RF Energy Harvesting.” IEEE Access 9: 97444–97456.

[jfds70871-bib-0100] Estimating the Burden of Foodborne Diseases . 2024. World Health Organization. 10.2471/BLT.14.148056 PMC4581658 26478611

[jfds70871-bib-0025] Eze, J. , Y. Duan , E. Eze , R. Ramanathan , and T. Ajmal . 2024. “Machine Learning‐Based Optimal Temperature Management Model for Safety and Quality Control of Perishable Food Supply Chain.” Scientific Reports 14, no. 1: 27228. 39516500 10.1038/s41598-024-70638-6 PMC11549301

[jfds70871-bib-0026] Fatorachian, H. , and K. Pawar . 2025. “Sustainable Cold Chain Management: An Evaluation of Predictive Waste Management Models.” Applied Sciences ‐ Basel 15, no. 2: 770.

[jfds70871-bib-0027] Feng, H. , J. Fan , Y. Ji , B. Glamuzina , and R. Ma . 2024. “Reliable Quality Traceability for Tilapia Cold Chain Using Blockchain and Machine Learning Techniques.” Journal of Food Process Engineering 47, no. 12: e70016.

[jfds70871-bib-0028] Feng, H. , Y. Fu , S. Huang , B. Glamuzina , and X. Zhang . 2023. “Novel Flexible Sensing Technology for Nondestructive Detection on Live Fish Health/Quality During Waterless and Low‐Temperature Transportation.” Biosensors and Bioelectronics 228: 115211. 36917894 10.1016/j.bios.2023.115211

[jfds70871-bib-0029] Feng, H. , M. Zhang , P. Liu , Y. Liu , and X. Zhang . 2020. “Evaluation of IoT‐Enabled Monitoring and Electronic Nose Spoilage Detection for Salmon Freshness During Cold Storage.” Foods 9, no. 11: 1579. 33143312 10.3390/foods9111579 PMC7692724

[jfds70871-bib-0030] Finmile . 2025. “The True ROI of AI in Logistics: Unlocking Intelligent, High–Impact Supply Chain Operations.” Accessed November 3, 2025. https://finmile.co/resources/the-true-roi-of-ai-in-logistics-unlocking-intelligent-high-impact-supply-chain-operations.

[jfds70871-bib-0095] Food Waste Index Report . 2024. Think Eat Save: Tracking Progress to Halve Global Food Waste 2024. United Nations Environment Programme.

[jfds70871-bib-0032] Fotopoulos, A. , P. Z. Lappas , and A. Melitsiotis . 2022. “The Edge‐Cloud Continuum in Wearable Sensing for Respiratory Analysis.” In Wearable Sensing and Intelligent Data Analysis for Respiratory Management, 241–271. Elsevier.

[jfds70871-bib-0033] Garlito, B. , M. A. Sentandreu , V. Yusà , M. Oliván , O. Pardo , and E. Sentandreu . 2023. “New Insights Into the Search of Meat Quality Biomarkers Assisted By Orbitrap Tribrid Untargeted Metabolite Analysis and Chemometrics.” Food Chemistry 407: 135173. 36527949 10.1016/j.foodchem.2022.135173

[jfds70871-bib-0034] Gillespie, J. , T. P. da Costa , X. Cama‐Moncunill , et al. 2023. “Real‐Time Anomaly Detection in Cold Chain Transportation Using IoT Technology.” Sustainability 15, no. 3: 2255.

[jfds70871-bib-0035] Goyal, M. , and Q. H. Mahmoud . 2024. “A Systematic Review of Synthetic Data Generation Techniques Using Generative AI.” Electronics 13, no. 17: 3509.

[jfds70871-bib-0036] Grecuccio, J. , E. Giusto , F. Fiori , and M. Rebaudengo . 2020. “Combining Blockchain and IoT: Food‐Chain Traceability and Beyond.” Energies 13, no. 15: 3820.

[jfds70871-bib-0037] Guo, N. , B. Qian , J. Na , R. Hu , and J.‐L. Mao . 2022. “A Three‐Dimensional Ant Colony Optimization Algorithm for Multi‐Compartment Vehicle Routing Problem Considering Carbon Emissions.” Applied Soft Computing 127: 109326.

[jfds70871-bib-0039] Haider, A. , R. Kazmi , T. Alam , et al. 2024. “IoT‐ Enabled Firmness Grades of Tomato in Cold Supply Chain Using Fusion of Whale Optimization Algorithm and Extreme Learning Machine.” IEEE Access 12: 52744–52758.

[jfds70871-bib-0040] Hassija, V. , V. Chamola , A. Mahapatra , et al. 2023. “Interpreting Black‐Box Models: A Review on Explainable Artificial Intelligence.” Cognitive Computation 16, no. 1: 45–74.

[jfds70871-bib-0041] Ho, S. M. , D. Ryan , M. Huang , and L. Harris . 2024. “Asia‐Pacific Regulations Keep Pace With Rapid Evolution of Artificial Intelligence Technology.” Accessed November 3, 2025.

[jfds70871-bib-0042] Hu, J. , D. Liu , Y. Zhu , et al. 2024. “Establishing A Maturity Prediction Model for Respiratory Fruits Via Ethylene‐Regulated Physiology: A Case Investigation of Avocado.” Food Bioscience 59: 104097.

[jfds70871-bib-0043] Hu, L. , C. Xiang , and C. Qi . 2021. “Optimization of VRR for Cold Chain with Minimum Loss Based on Actual Traffic Conditions.” Wireless Communications and Mobile Computing 2021: 2930366.

[jfds70871-bib-0044] Huang, W. , X. Wang , J. Zhang , J. Xia , and X. Zhang . 2023a. “Improvement of Blueberry Freshness Prediction Based on Machine Learning and Multi‐Source Sensing in the Cold Chain Logistics.” Food Control 145: 109496.

[jfds70871-bib-0045] Huang, W. , J. Xia , X. Wang , Q. Zhao , M. Zhang , and X. Zhang . 2023b. “Improvement of Non‐Destructive Detection of Lamb Freshness Based on Dual‐Parameter Flexible Temperature‐Impedance Sensor.” Food Control 153: 109963.

[jfds70871-bib-0046] ISO 23412:2020 . 2020. Indirect, Temperature‐Controlled Refrigerated Delivery Services – Requirements for Refrigerated Transport of Temperature‐Sensitive Goods. 2020. International Organization for Standardization.

[jfds70871-bib-0047] Jackson, I. , J. Namdar , M. J. Saenz , R. A. Elmquist III , and L. R. D. Novoa . 2024. “Revolutionize Cold Chain: An AI/ML Driven Approach To Overcome Capacity Shortages.” International Journal of Production Research 63, no. 6: 2190–2212.

[jfds70871-bib-0048] Kang, K. , S. Belkhale , G. Kahn , P. Abbeel , and S. Levine . 2019. “Generalization through Simulation: Integrating Simulated and Real Data into Deep Reinforcement Learning for Vision‐Based Autonomous Flight.” In 2019 International Conference on Robotics and Automation (ICRA). 6008–6014. IEEE.

[jfds70871-bib-0049] Kanjilal, R. , J. E. Saenz , and I. Uysal . 2025. “Large‐Scale Data‐Driven Uniformity Analysis and Sensory Prediction of Commercial Banana Ripening Process.” Postharvest Biology and Technology 219: 113203.

[jfds70871-bib-0050] Kim, T. H. , J. H. Kim , J. Y. Kim , and S. E. Oh . 2022. “Egg Freshness Prediction Model Using Real‐Time Cold Chain Storage Condition Based on Transfer Learning.” Foods 11, no. 19: 3082. 36230158 10.3390/foods11193082 PMC9564046

[jfds70871-bib-0051] Kreuzer, T. , P. Papapetrou , and J. Zdravkovic . 2024. “Artificial Intelligence in Digital Twins‐A Systematic Literature Review.” Data & Knowledge Engineering 151: 102304.

[jfds70871-bib-0052] Ktenioudaki, A. , C. P. O'Donnell , J. P. Emond , and M. C. do Nascimento Nunes . 2021. “Blueberry Supply Chain: Critical Steps Impacting Fruit Quality and Application of a Boosted Regression Tree Model To Predict Weight Loss.” Postharvest Biology and Technology 179: 111590.

[jfds70871-bib-0053] Kulkarni, A. , Y. Wang , M. Gopinath , D. Sobien , A. Rahman , and F. A. Batarseh . 2024. “A Review of Cybersecurity Incidents in the Food and Agriculture Sector.” Journal of Agriculture and Food Research 23: 102245.

[jfds70871-bib-0054] Kumar, S. , and S. Sharma . 2024. “Intelligent Transportation Storage Condition Assessment System for Fruits And Vegetables Supply Chain Using Internet of Things Enabled Sensor Network.” International Journal of System Assurance Engineering and Management, 1–15.

[jfds70871-bib-0055] K Kumari, R. 2025. “IoT Integration in Pharmaceuticals: Opportunities for Real‐Time Monitoring in Cold Chains.” Journal of Supply Chain & Digitalization Research 2, no. 1: 12–29.

[jfds70871-bib-0056] Lau, H. , Y. P. Tsang , D. Nakandala , and C. K. M. Lee . 2021. “Risk Quantification in Cold Chain Management: A Federated Learning‐Enabled Multi‐Criteria Decision‐Making Methodology.” Industrial Management & Data Systems 121, no. 7: 1684–1703.

[jfds70871-bib-0057] Lim, M. K. , Y. Li , C. Wang , and M.‐L. Tseng . 2022. “Prediction of Cold Chain Logistics Temperature Using A Novel Hybrid Model Based on the Mayfly Algorithm and Extreme Learning Machine.” Industrial Management & Data Systems 122, no. 3: 819–840.

[jfds70871-bib-0058] Liu, G. , Q. Liu , H. Guo , M. Xiang , and J. Sang . 2024a. “Optimization of “vehicle‐UAV” Joint Distribution Routing for Cold Chain Logistics Considering Risk of Epidemic Spreading and Green Cost.” PLoS ONE 19, no. 6: e0306127. 38924055 10.1371/journal.pone.0306127 PMC11207173

[jfds70871-bib-0059] Liu, Z. , Y. Li , J. Xu , and D. Bai . 2024b. “Multi‐Compartment Electric Vehicle Routing Problem for Perishable Products.” International Journal of Crowd Science 8, no. 1: 38–48.

[jfds70871-bib-0060] Loisel, J. , S. Duret , A. Cornuéjols , et al. 2021. “Cold Chain Break Detection and Analysis: Can Machine Learning Help?.” Trends in Food Science & Technology 122: 391–399.

[jfds70871-bib-0062] Maheswaran, S. , R. Gomathi , S. Sathesh , et al. 2024. “Intelligent Cold Chain Security: Nano Power Temperature Sensors, ESP32 and Telegram Bot Integration for Temperature Assurance and Environmental Harm Prevention.” Journal of Environmental Nanotechnology 13, no. 1: 17–25.

[jfds70871-bib-0063] Meng, X. , R. Xie , J. Liao , X. Shen , and S. Yang . 2024. “A Cost‐Effective Over‐Temperature Alarm System for Cold Chain Delivery.” Journal of Food Engineering 368: 111914.

[jfds70871-bib-0064] Minhar, E. 2025. “The Hidden Crisis: Temperature‐Related Pharma Losses.” Accessed November 3, 2025. https://sensos.io/resources/cold-chain-pharma/the-hidden-crisis-temperature%E2%80%91related-pharma-losses/.

[jfds70871-bib-0065] Mmereki, D. , V. E. David , and A. H. Wreh Brownell . 2023. “The Management and Prevention of Food Losses and Waste in Low‐ and Middle‐Income Countries: A Mini‐Review in the Africa Region.” Waste Management & Research: The Journal for a Sustainable Circular Economy 42, no. 4: 287–307. 10.1177/0734242X231184444 PMC10983775 37533307

[jfds70871-bib-0066] Montes, J. B. , S. Z. Fernandez , and V. D. Casas . 2024. “Internet of Things in Energy‐Sensitive Processes: Application in a Refrigerated Warehouse.” IEEE Access 12: 76257–76276.

[jfds70871-bib-0067] Mrabet, H. , S. Belguith , A. Alhomoud , and A. Jemai . 2020. “A Survey of IoT Security Based on a Layered Architecture of Sensing and Data Analysis.” Sensors 20, no. 13: 3625. 32605178 10.3390/s20133625 PMC7374330

[jfds70871-bib-0093] M Tatar, A. 2024. “Effects of Intensive and Conventional Farming on Oxidative Stress and Meat Quality Biomarkers in Holstein and Simmental Cattle.” Scientific Reports 14, no. 1: 26197. 39478177 10.1038/s41598-024-78087-x PMC11526110

[jfds70871-bib-0068] Mustafa, M. F. M. S. , N. Navaranjan , and A. Demirovic . 2024. “Food Cold Chain Logistics and Management: A Review of Current Development and Emerging Trends.” Journal of Agriculture and Food Research 18: 101343.

[jfds70871-bib-0069] Nakandala, D. , H. Lau , and J. Zhang . 2016. “Cost‐Optimization Modelling for Fresh Food Quality and Transportation.” Industrial Management and Data Systems 116, no. 3: 564–583.

[jfds70871-bib-0070] Nong, K. , H. Zhang , and Z. Liu . 2024. “Comparative Study of Different Machine Learning Models for Heat Transfer Performance Prediction of Evaporators in Modular Refrigerated Display Cabinets.” Energies 17, no. 23: 6189.

[jfds70871-bib-0071] Obaidat, M. , J. Brown , S. Obeidat , and M. Rawashdeh . 2020. “A Hybrid Dynamic Encryption Scheme for Multi‐Factor Verification: A Novel Paradigm for Remote Authentication.” Sensors 20, no. 15: 4212. 32751189 10.3390/s20154212 PMC7435875

[jfds70871-bib-0072] Ogawa, N. N. , G. L. Silva , A. P. A. d. C. Barbon , et al. 2024. “Animal Welfare Assessment and Meat Quality through Assessment of Stress Biomarkers in Fattening Pigs with and without Visible Damage during Slaughter.” Animals 14, no. 5: 700. 38473085 10.3390/ani14050700 PMC10931360

[jfds70871-bib-0073] Oluwafemi, I. O. , T. Clement , O. S. Adanigbo , T. P. Gbenle , and B. I. Adekunle . 2024. “Investigating the Trade‐Off between Data Granularity and Consumer Trust in Federated Marketing Analytics Using Differential Privacy Techniques.” International Journal of Scientific Research in Science and Technology 11, no. 5: 701–717.

[jfds70871-bib-0074] Onoufriou, G. , M. Hanheide , and G. Leontidis . 2021. “EDLaaS: Fully Homomorphic Encryption Over Neural Network Graphs for Vision and Private Strawberry Yield Forecasting.” Sensors 22, no. 21: 8124. 10.3390/s22218124 PMC9658784 36365823

[jfds70871-bib-0075] Papatsiros, V. G. , G. Maragkakis , and G. I. Papakonstantinou . 2024. “Stress Biomarkers in Pigs: Current Insights and Clinical Application.” Veterinary Sciences 11, no. 12: 640. 39728980 10.3390/vetsci11120640 PMC11680096

[jfds70871-bib-0076] Park, Y. , S. Kim , and K. Ryu . 2023. “Prediction of Refrigerated Vehicle Environment for Optimization of Cold‐Chain Logistics.” ICIC Express Letters, Part B: Applications 14, no. 2: 193–199.

[jfds70871-bib-0077] Peng, J. , R. Xiao , C. Wu , et al. 2024. “Characterization of the Prevalence of Salmonella in Different Retail Chicken Supply Modes Using Genome‐Wide and Machine‐Learning Analyses.” Food Research International 191: 114654. 39059904 10.1016/j.foodres.2024.114654

[jfds70871-bib-0078] Pessoa, J. , C. McAloon , M. Rodrigues da Costa , E. Garcia Manzanilla , T. Norton , and L. Boyle . 2021. “Adding Value to Food Chain Information: Using Data on Pig Welfare and Antimicrobial Use On‐Farm To Predict Meat Inspection Outcomes.” Porcine Health Management 7, no. 1: 55. 34649629 10.1186/s40813-021-00234-x PMC8518164

[jfds70871-bib-0079] Qian, J. , Q. Yu , L. Jiang , H. Yang , and W. Wu . 2022. “Food Cold Chain Management Improvement: A Conjoint Analysis on COVID‐19 and Food Cold Chain Systems.” Food Control 137: 108940. 35261485 10.1016/j.foodcont.2022.108940 PMC8890692

[jfds70871-bib-0080] Radanliev, P. , D. De Roure , R. Nicolescu , M. Huth , and O. Santos . 2021. “Digital Twins: Artificial Intelligence and the IoT Cyber‐Physical Systems in Industry 4.0.” International Journal of Intelligent Robotics and Applications 6, no. 1: 171–185.

[jfds70871-bib-0081] Ramirez‐Asis, E. , A. Bhanot , V. Jagota , et al. 2022. “Smart Logistic System for Enhancing the Farmer‐Customer Corridor in Smart Agriculture Sector Using Artificial Intelligence.” Journal of Food Quality 2022: 7486974.

[jfds70871-bib-0082] Roduit, B. , C. A. Luyet , M. Hartmann , et al. 2019. “Continuous Monitoring of Shelf Lives of Materials by Application of Data Loggers With Implemented Kinetic Parameters.” Molecules 24, no. 12: 2217. 31200557 10.3390/molecules24122217 PMC6631491

[jfds70871-bib-0083] Romanello, R. , and V. Veglio . 2022. “Industry 4.0 in Food Processing: Drivers, Challenges and Outcomes.” British Food Journal 124, no. 13: 375–390.

[jfds70871-bib-0084] Russel, S. , and P. Norvig . 2009. Artificial Intelligence: A Modern Approach. Prentice Hall Press.

[jfds70871-bib-0085] Schöning, J. , A. Riechmann , and H.‐J. Pfisterer . 2022. “AI for Closed‐Loop Control Systems: New Opportunities for Modeling, Designing, and Tuning Control Systems.” In *Proceedings of the 2022 14th International Conference on Machine Learning and Computing*, *ICMLC '22*, 318–323. Association for Computing Machinery.

[jfds70871-bib-0024] Şekeroǧlu, A. , Y. Şentürk , B. Tainika , M. Duman , and A. Akyol . 2024. “The Impact of Laying Hen Age, Egg‐Laying Time, Cage Tier, and Cage Direction on Egg Quality Traits in Hens in an Enriched Cage System.” Brazilian Journal of Poultry Science 26, no. 2: 1–9.

[jfds70871-bib-0086] Sharma, K. 2017. “Automation Strategies.” In Overview of Industrial Process Automation, 53–74. Elsevier.

[jfds70871-bib-0087] Sharma, S. , A. Malik , C. Sharma , I. Batra , M. S. Kaswan , and J. A. Garza‐Reyes . 2023. “Adoption of Industry 4.0 in Different Sectors: A Structural Review Using Natural Language Processing.” International Journal on Interactive Design and Manufacturing 18, no. 8: 6069–6091.

[jfds70871-bib-0088] Shi, J. 2024. “Optimization of Frozen Goods Distribution Logistics Network Based on K‐Means Algorithm and Priority Classification.” Scientific Reports 14, no. 1: 22477. 39341884 10.1038/s41598-024-72723-2 PMC11438974

[jfds70871-bib-0089] Shih, C.‐W. , and C.‐H. Wang . 2016. “Integrating Wireless Sensor Networks with Statistical Quality Control To Develop A Cold Chain System in Food Industries.” Computer Standards and Interfaces 45: 62–78.

[jfds70871-bib-0090] Singh, A. K. , and Z. Raza . 2023. “A Framework for IoT and Blockchain Based Smart Food Chain Management System.” Concurrency and Computation: Practice and Experience 35, no. 4: e7526.

[jfds70871-bib-0091] Singh, R. , and S. S. Gill . 2023. “Edge AI: A Survey.” Internet of Things and Cyber‐Physical Systems 3: 71–92.

[jfds70871-bib-0092] Tang, J. , Y. Zou , R. Xie , B. Tu , and G. Liu . 2021. “Compact Supervisory System for Cold Chain Logistics.” Food Control 126: 108025.

[jfds70871-bib-0094] Uhlig, E. , A. Sadzik , M. Strenger , A.‐M. Schneider , and M. Schmid . 2025. “Food Wastage Along The Global Food Supply Chain and the Impact of Food Packaging.” Journal of Consumer Protection and Food Safety 20, no. 1: 5–17.

[jfds70871-bib-0031] U.S. Food and Drug Administration . 2022. “The FDA Moves into Third Phase of Artificial Intelligence Imported Seafood Pilot Program.” Accessed November 3, 2025. https://www.fda.gov/food/hfp-constituent-updates/fda-moves-third-phase-artificial-intelligence-imported-seafood-pilot-program.

[jfds70871-bib-0096] Voigt, P. , and A. von dem Bussche . 2024. The EU General Data Protection Regulation (GDPR). A Practical Guide. Springer Nature.

[jfds70871-bib-0061] Voola, P. , and M. Rajini . 2025. “Developing an IoT and ML‐Driven Platform for Fruit Ripeness Evaluation And Spoilage Detection: A Case Study on Bananas.” e‐Prime ‐ Advances in Electrical Engineering, Electronics and Energy 11: 100896.

[jfds70871-bib-0097] Walters, J. , D. Dey , D. Bhaumik , and S. Horsman . 2023. “Complying With the EU AI Act.” arXiv:2307.10458.

[jfds70871-bib-0098] Wang, B. , Y. Li , K. Liu , et al. 2024. “Intelligent Evaluation and Dynamic Prediction of Oysters Freshness with Electronic Nose Non‐Destructive Monitoring and Machine Learning.” Biosensors‐Basel 14, no. 10: 502. 39451715 10.3390/bios14100502 PMC11506465

[jfds70871-bib-0099] Wang, X. 2023. “An Analysis of Temperature Control Management in the Pharmaceutical Supply Chain.” Logistics & Supply Chain Management 6: 45–64.

[jfds70871-bib-0101] Xia, J. , W. Huang , X. Wang , Z. Zhu , M. Zhang , and X. Zhang . 2023. “Flexible Sensing Technology for Fruit Quality Control in the Cold Chain: Characterization, Application, and Improvement.” Food Control 154: 109976.

[jfds70871-bib-0102] Xing, T. , F. Gao , R. K. Tume , G. Zhou , and X. Xu . 2018. “Stress Effects on Meat Quality: A Mechanistic Perspective.” Comprehensive Reviews in Food Science and Food Safety 18, no. 2: 380–401. 33336942 10.1111/1541-4337.12417

[jfds70871-bib-0103] Xu, J. , H. Li , and Q. Zhang . 2024. “Adaptive Control for Circulating Cooling Water System Using Deep Reinforcement Learning.” PLoS ONE 19: e0307767. 39047030 10.1371/journal.pone.0307767 PMC11268623

[jfds70871-bib-0104] Yang, L. , Y. Gao , Y. Sun , and J. Li . 2024. “Two‐Phase Hybrid Search Algorithm for Time‐Dependent Cold Chain Logistics Route Considering Carbon Emission and Traffic Congestion.” IEEE Access 12: 95128–95151.

[jfds70871-bib-0105] Yu, M. , H. Zhang , J. Ma , X. Duan , S. Kang , and J. Li . 2025. “Cold Chain Logistics Supervision of Agricultural Products Supported Using Internet of Things Technology.” IEEE Internet of Things Journal 12, no. 4: 3502–3511.

[jfds70871-bib-0106] Yuan, B. , F. Tao , Y. Qin , Q. Chen , and Y. Su . 2025. “Can Policy Achieve the Goal of Cold Chain Logistics Sustainable Development?.” Transportation Research Part D: Transport and Environment 140: 104607.

[jfds70871-bib-0107] Zakeri, A. , M. Saber , O. K. Hussain , and E. Chang . 2018. “An Early Detection System for Proactive Management of Raw Milk Quality: An Australian Case Study.” IEEE Access 6: 64333–64349.

[jfds70871-bib-0108] Zhang, B. , J. Xu , X. Wang , Z. Zhao , S. Chen , and X. Zhang . 2023. “Research on the Construction of Grain Food Multi‐Chain Blockchain Based on Zero‐Knowledge Proof.” Foods 12, no. 8: 1600. 37107395 10.3390/foods12081600 PMC10138098

[jfds70871-bib-0109] Zhang, D. , L. Zhang , B. Cong , J. Hu , and X. Zhang . 2024a. “Multi‐Frequency Bioimpedance Combined with Machine Learning for Frozen‐Thawed Quality Evaluation of Atlantic Salmon.” Journal of Food Engineering 382: 112195.

[jfds70871-bib-0110] Zhang, L. , M. Zhang , A. S. Mujumdar , C. Wu , and D. Wang . 2024b. “Advanced Model Predictive Control Strategies For Nondestructive Monitoring Quality of Fruit and Vegetables During Supply Chain Processes.” Computers and Electronics in Agriculture 225: 109262.

[jfds70871-bib-0111] Zhang, Y. , Y. Liu , Z. Jiong , X. Zhang , B. Li , and E. Chen . 2021. “Development and Assessment of Blockchain‐IoT‐Based Traceability System for Frozen Aquatic Product.” Journal of Food Process Engineering 44, no. 5: e13669.

[jfds70871-bib-0112] Zhong, X. , M. Zhang , T. Tang , B. Adhikari , and Y. Ma . 2023. “Advances in Intelligent Detection, Monitoring, and Control for Preserving the Quality of Fresh Fruits and Vegetables in the Supply Chain.” Food Bioscience 56: 103350.

[jfds70871-bib-0113] Zhou, Y. , and X. Chen . 2025. “Edge Intelligence: Edge Computing for 5G and the Internet of Things.” Future Internet 17, no. 3: 101.

[jfds70871-bib-0114] Zou, Y. , J. Wu , X. Wang , K. Morales , G. Liu , and A. Manzardo . 2023. “An Improved Artificial Neural Network Using Multi‐Source Data To Estimate Food Temperature During Multi‐Temperature Delivery.” Journal of Food Engineering 351: 111518.

[jfds70871-bib-0115] Zou, Z. , Q. Chen , I. Uysal , and L. Zheng . 2014. “Radio Frequency Identification Enabled Wireless Sensing for Intelligent Food Logistics.” Philosophical Transactions of the Royal Society A: Mathematical, Physical and Engineering Sciences 372, no. 2017: 20130313. 10.1098/rsta.2013.0313 24797140

