# Supplementary material for: Enhancing Food Safety in the Cold Chain Through Internet of Things and Artificial Intelligence
Source: J Food Sci. 2026 Feb 16;91(2):e70871. doi: 10.1111/1750-3841.70871 (PMC12910151; doi:10.1111/1750-3841.70871)
Supplement: Supplementary file 1 — Table S1. Summary of sensors used in each study. [file JFDS-91-0-s001.pdf]

Supporting Information for Enhancing Food Safety in the Cold Chain through Internet of Things and Artificial Intelligence

WAGNER A. MÜLLER, SANDRO BINSFELD FERREIRA, SUSE BOTELHO DA SILVA*

* Corresponding author: susebs@unisinos.br

1. RESUME OF STUDIES AND APPLIED SENSORS

Sensors for alcohol and LPG [1], fire [2], NH_3 [3], weight [4], pH, and air quality [5] were not added to the table because they appeared only once in the reviewed studies.

Table S1. Summary of sensors used in each study. Abbreviations: T – Temperature, RH – relative humidity, P – pressure, L – light level, D – door sensor, V – vibration, and C – camera.

Study	T	RH	GPS	C_2H_4	H_2	CH_4	O_2	CO	CO_2	H_2S	L	P	D	V	C
6	X	X							X		X				
3	X	X		X					X						
5	X	X													
7	X	X	X												X
8	X	X	X											X	
9	X	X	X												
10	X	X									X				
11	X	X													
12	X								X						
13	X	X	X						X		X			X	
14	X	X					X								
15	X	X					X		X						
16	X	X													
17	X	X				X		X							
18	X	X	X												
1	X	X			X	X		X				X			
2	X	X												X	
4	X	X													
19	X	X		X					X		X				
20	X	X	X												
21	X	X	X												
22	X	X													X
23	X	X		X			X		X						
24	X	X		X					X						
25	X	X													
26	X														
27	X														
28	X	X													
29	X	X	X	X							X				
30	X	X				X									
31	X														
32	X														
33	X														
34	X		X										X		
35	X										X	X			
36	X														
37	X	X	X										X		
38	X	X													

Continued on next page

(Continued from previous page)

Study	T	RH	GPS	C_2H_4	H_2	CH_4	O_2	CO	CO_2	H_2S	L	P	D	V	C
[39]	X		X												
[40]	X	X					X		X	X					
[41]	X	X										X			
[42]	X														
[43]	X	X													
[44]	X	X													
[45]	X	X	X												
[46]	X	X			X				X	X					
[47]	X	X					X								X
[48]	X														
[49]	X	X	X												
[50]	X	X													
[51]	X	X	X												
[52]	X	X	X				X		X						
[53]	X	X	X								X				
[54]	X	X	X										X	X	
[55]	X	X					X		X						
[56]	X	X	X												
[57]	X	X													
[58]	X	X													

REFERENCES

[1] A. Popa, M. Hnatiuc, M. Paun, O. Geman, D. J. Hemanth, D. Dorcea, L. H. Son, and S. Ghita, "An intelligent iot-based food quality monitoring approach using low-cost sensors," Symmetry **11** (2019).

[2] K. Mohanraj, S. Vijayalakshmi, N. Balaji, R. Chithrakkannan, and R. Karthikeyan, "Smart warehouse monitoring using iot," Int. J. Eng. Adv. Technol. **8**, 3597 – 3600 (2019).

[3] A. N. Damdam, L. O. Ozay, C. K. Ozcan, A. Alzahrani, R. Helabi, and K. N. Salama, "Iot-enabled electronic nose system for beef quality monitoring and spoilage detection," Foods **12** (2023).

[4] S. Vijayashaarathi, K. Saranya, K. E. Viswesvaran, and K. S. S. Sruthi, "Iot-based smart storage system using peltier technology," J. Popul. Ther. Clin. Pharmacol. **30**, E322–E331 (2023).

[5] S. Kumar and S. Sharma, "Intelligent transportation storage condition assessment system for fruits and vegetables supply chain using internet of things enabled sensor network," Int. J. Syst. Assur. Eng. Manag. (2024).

[6] H. Afreen and I. S. Bajwa, "An iot-based real-time intelligent monitoring and notification system of cold storage," IEEE Access **9**, 38236 – 38253 (2021).

[7] G. Alfian, M. Syafrudin, and J. Rhee, "Real-time monitoring system using smartphone-based sensors and nosql database for perishable supply chain," Sustain. (Switzerland) **9** (2017).

[8] A. Haider, R. Kazmi, T. Alam, R. N. Bashir, H. Nobanee, A. R. Khan, and Aqsa, "Iot- enabled firmness grades of tomato in cold supply chain using fusion of whale optimization algorithm and extreme learning machine," IEEE Access **12**, 52744–52758 (2024).

[9] G. Baralla, A. Pinna, R. Tonelli, M. Marchesi, and S. Ibba, "Ensuring transparency and traceability of food local products: A blockchain application to a smart tourism region," Concurr. Comput. & Exp. **33** (2021).

[10] Y. P. Tsang, K. L. Choy, C. H. Wu, G. T. S. Ho, C. H. Y. Lam, and P. S. Koo, "An internet of things (iot)-based risk monitoring system for managing cold supply chain risks," Ind. Manag. & Data Syst. **118**, 1432–1462 (2018).

[11] G. Alfian, M. Syafrudin, U. Farooq, M. R. Ma'arif, M. A. Syaekhoni, N. L. Fitriyani, J. Lee, and J. Rhee, "Improving efficiency of rfid-based traceability system for perishable food by utilizing iot sensors and machine learning model," Food Control. **110** (2020).

[12] A. Abougharib, M. Awad, and M. Ndiaye, "Remaining shelf-life estimation of fresh fruits and vegetables during transportation," IEEE Access **11**, 8845 – 8859 (2023).

[13] J. Garrido-López, M. Jiménez-Buendía, A. Toledo-Moreo, J. Giménez-Gallego, and R. Torres-Sánchez, "Monitoring perishable commodities using cellular iot: An intelligent real-time conditions tracker design," Appl. Sci. (Switzerland) **14** (2024).

[14] S. Alsubai, A. Alqahtani, A. Alanazi, and M. Bhatia, "Decision-tree-assisted intelligent framework for food quality analysis," IEEE Internet Things J. **11**, 30800 – 30807 (2024).

[15] H. Feng, J. Fan, Y. Ji, B. Glamuzina, and R. Ma, "Reliable quality traceability for tilapia cold chain using blockchain and machine learning techniques," J. Food Process. Eng. **47** (2024).
[16] L. K. Baghel, R. Raina, S. Kumar, and L. Catarinucci, "Iot-based integrated sensing and logging solution for cold chain monitoring applications," IEEE J. Radio Freq. Identif. **8**, 837 – 846 (2024).
[17] S. Maheswaran, R. Gomathi, S. Sathesh, S. Poovizhi, R. Ridhish, S. Nanthakkumaran, and M. Chinnadurai, "Intelligent cold chain security: Nano power temperature sensors, esp32 and telegram bot integration for temperature assurance and environmental harm prevention," J. Environ. Nanotechnol. **13**, 17 – 25 (2024).
[18] J. Gillespie, T. P. da Costa, X. Cama-Moncunill, T. Cadden, J. Condell, T. Cowderoy, E. Ramsey, F. Murphy, M. Kull, R. Gallagher, and R. Ramanathan, "Real-time anomaly detection in cold chain transportation using iot technology," Sustain. (Switzerland) **15** (2023).
[19] M. Mohammed, K. Riad, and N. Alqahtani, "Design of a smart iot-based control system for remotely managing cold storage facilities," Sensors **22** (2022).
[20] A. I. Sourav, N. D. Lynn, and Suyoto, "Smart monitoring system design for perishable food supply chain management based on iot in bangladesh," Int. J. Adv. Sci. Technol. **29**, 1069 – 1079 (2020).
[21] O. Urbano, A. Perles, C. Pedraza, S. Rubio-Arraez, M. L. Castelló, M. D. Ortola, and R. Mercado, "Cost-effective implementation of a temperature traceability system based on smart rfid tags and iot services," Sensors (Switzerland) **20** (2020).
[22] K. Nair, B. Sekhani, K. Shah, and S. Karamchandani, "Expiry prediction and reducing food wastage using iot and ml," Int. J. Electr. Comput. Eng. Syst. **12**, 155 – 162 (2021).
[23] R. Wason, P. Arora, A. Tomar, and D. Arora, "A novel, low-cost, smart iot based framework for fruit and vegetable quality detection during transit in india," Int. J. Inf. Technol. (Singapore) **15**, 1509 – 1519 (2023).
[24] S. Bagchi, M. Jenamani, and A. Routray, "A novel data transformation for improving predictive accuracy of online missing value imputation during reefer container monitoring," IEEE Sensors J. **24**, 38286–38297 (2024).
[25] M. T. Seman, M. Abdullah, and M. Ishak, "Monitoring temperature, humidity and controlling system in industrial fixed room storage based on iot," J. Eng. Sci. Technol. **15**, 3588 – 3600 (2020).
[26] B. Roduit, C. A. Luyet, M. Hartmann, P. Folly, A. Sarbach, A. Dejeaifve, R. Dobson, N. Schroeter, O. Vorlet, M. Dabros, and R. Baltensperger, "Continuous monitoring of shelf lives of materials by application of data loggers with implemented kinetic parameters," Molecules **24** (2019).
[27] J. Ramírez-Faz, L. M. Fernández-Ahumada, E. Fernández-Ahumada, and R. López-Luque, "Monitoring of temperature in retail refrigerated cabinets applying iot over open-source hardware and software," Sensors (Switzerland) **20** (2020).
[28] U. Ramanathan, R. Ramanathan, A. Adefisan, T. Da Costa, X. Cama-Moncunill, and G. Samriya, "Adapting digital technologies to reduce food waste and improve operational efficiency of a frozen food company—the case of yumchop foods in the uk," Sustain. (Switzerland) **14** (2022).
[29] J. Qian, Q. Yu, L. Jiang, H. Yang, and W. Wu, "Food cold chain management improvement: A conjoint analysis on covid-19 and food cold chain systems," Food Control. **137** (2022).
[30] B. P. Nemade, K. Shah, B. Marakarkandy, K. Shah, B. C. Surve, and R. K. Nagra, "An efficient iot-based automated food waste management system with food spoilage detection," Int. J. Intell. Syst. Appl. Eng. **12**, 434 – 449 (2024).
[31] P. Morillo, J. M. Orduña, M. Fernández, and I. García-Pereira, "Comparison of wsn and iot approaches for a real-time monitoring system of meal distribution trolleys: A case study," Futur. Gener. Comput. Syst. **87**, 242–250 (2018).
[32] J. B. Montes, S. Z. Fernandez, and V. D. Casas, "Internet of things in energy-sensitive processes: Application in a refrigerated warehouse," IEEE Access **12**, 76257 – 76276 (2024).
[33] A. M. J. Marindra, B. M. Pratama, and D. J. Suroso, "Non-invasive frozen meat monitoring system using uhf rfid tag antenna-based sensing and rssi," Int. J. on Adv. Sci. Eng. Inf. Technol. **13**, 1–7 (2023).
[34] S. Khaengkarn, K. Nonkeaw, T. Wonglomklang, and J. Srisertpol, "Real-time tracking and environmental monitoring system for ice trucks using iot techniques," WSEAS Transactions on Inf. Sci. Appl. **19**, 297 – 301 (2022).
[35] R. Karthikeyan and B. Raghu, "Design of event management system for smart retail stores with iot edge," Int. J. Eng. Trends Technol. **68**, 81 – 88 (2020).
[36] S. Kananian, J. Rho, C. Chen, S. Mirjalili, A. Daus, M. gu Kim, S. Niu, E. Pop, H.-S. P. Wong, Z. Bao, A. Mani, and A. S. Poon, "A disposable reader-sensor solution for wireless temperature logging," Device **1**, 100183 (2023).
[37] Q. Jinping, "Application of wireless sensor on intelligent monitoring in logistics refrigerator car transportation," Sensors Transducers **160**, 250 – 255 (2013).
[38] T. Inthasuth, W. Boonsong, and T. Naemsai, "Implementation of zigbee and nb-iot networks in cooling monitoring systems for pelter-based mini refrigerators," Przeglad Elektrotechniczny **1**, 126 – 132 (2023).
[39] J. Grecuccio, E. Giusto, F. Fiori, and M. Rebaudengo, "Combining blockchain and iot: Food-chain traceability and beyond," Energies **13** (2020).
[40] H. Feng, M. Zhang, P. Liu, Y. Liu, and X. Zhang, "Evaluation of iot-enabled monitoring and electronic nose spoilage detection for salmon freshness during cold storage," Foods **9** (2020).
[41] H.-D. Do, D.-E. Kim, M. B. Lam, and W.-Y. Chung, "Self-powered food assessment system using lstm network and 915 mhz rf energy harvesting," IEEE Access **9**, 97444 – 97456 (2021).

[42] H. Chu, G. Wu, J. Chen, F. Fei, J. D. Mai, and W. J. Li, "Design and simulation of self-powered radio frequency identification (rfid) tags for mobile temperature monitoring," Sci. China Technol. Sci. **56**, 1 – 7 (2013).
[43] Y.-Y. Chen, Y.-J. Wang, and J.-K. Jan, "A novel deployment of smart cold chain system using 2g-rfid-sys," J. Food Eng. **141**, 113 – 121 (2014).
[44] A. Alshdadi, S. Kamel, E. Alsolami, M. D. Lytras, and S. Boubaker, "An iot smart system for cold supply chain storage and transportation management," Eng. Technol. Appl. Sci. Res. **14**, 13167 – 13172 (2024).
[45] H. J. Jara Ochoa, R. Pena, Y. Ledo Mezquita, E. Gonzalez, and S. Camacho-Leon, "Comparative analysis of power consumption between mqtt and http protocols in an iot platform designed and implemented for remote real-time monitoring of long-term cold chain transport operations," Sensors **23** (2023).
[46] J. Xu, R. Ma, S. Stankovski, X. Liu, and X. Zhang, "Intelligent dynamic quality prediction of chilled chicken with integrated iot flexible sensing and knowledge rules extraction," Foods **11** (2022).
[47] M. Bhatia and A. Manocha, "Cognitive framework of food quality assessment in iot-inspired smart restaurants," IEEE Internet Things J. **9**, 6350–6358 (2022).
[48] C.-W. Shih and C.-H. Wang, "Integrating wireless sensor networks with statistical quality control to develop a cold chain system in food industries," Comput. Standards Interfaces **45**, 62 – 78 (2016).
[49] Y. Tsang, K. Choy, C. Wu, G. Ho, H. Lam, and V. Tang, "An intelligent model for assuring food quality in managing a multi-temperature food distribution centre," Food Control. **90**, 81–97 (2018).
[50] Y. Tsang, C. Wu, H. Lam, K. Choy, and G. Ho, "Integrating internet of things and multi-temperature delivery planning for perishable food e-commerce logistics: a model and application," Int. J. Prod. Res. **59**, 1534 – 1556 (2021).
[51] H. Luo, M. Zhu, S. Ye, H. Hou, Y. Chen, and L. Bulysheva, "An intelligent tracking system based on internet of things for the cold chain," Internet Res. **26**, 435–445 (2016).
[52] X. Li, L. Yang, Y. Duan, Z. Wu, and X. Zhang, "Developing a real-time monitoring traceability system for cold chain of tricholoma matsutake," Electronics **8** (2019).
[53] I. Sergi, T. Montanaro, F. L. Benvenuto, and L. Patrono, "A smart and secure logistics system based on iot and cloud technologies," Sensors **21** (2021).
[54] Y. Zhang, Y. Liu, Z. Jiong, X. Zhang, B. Li, and E. Chen, "Development and assessment of blockchain-iot-based traceability system for frozen aquatic product," J. Food Process. Eng. **44** (2021).
[55] H. Feng, W. Wang, B. Chen, and X. Zhang, "Evaluation on frozen shellfish quality by blockchain based multi-sensors monitoring and svm algorithm during cold storage," IEEE Access **8**, 54361–54370 (2020).
[56] A. K. Singh and Z. Raza, "A framework for iot and blockchain based smart food chain management system," Concurr. Comput. Pract. Exp. **35** (2023).
[57] M. Yu, H. Zhang, J. Ma, X. Duan, S. Kang, and J. Li, "Cold chain logistics supervision of agricultural products supported using internet of things technology," IEEE Internet Things J. **12**, 3502–3511 (2025).
[58] Z. Wu, X. Chen, Z. Wan, J. Chi, R. Zhang, M. Wang, D. Song, and X. Xiao, "Self-powered wireless flexible sensing for food storage based on triboelectric-electromagnetic generator," Mater. Today Sustain. **26** (2024).
